# Apoptotic signaling by TNFR1 is inhibited by the α2-6 sialylation, but not α2-3 sialylation, of the TNFR1 *N*-glycans

**DOI:** 10.1016/j.jbc.2024.108043

**Published:** 2024-11-29

**Authors:** Jihye Hwang, Tejeshwar C. Rao, Jiahui Tao, Bingdong Sha, Yoshiki Narimatsu, Henrik Clausen, Alexa L. Mattheyses, Susan L. Bellis

**Affiliations:** 1 Department of Cell, Developmental and Integrative Biology, University of Alabama at Birmingham, Birmingham, Alabama, USA; 2 Copenhagen Center for Glycomics, Department of Cellular and Molecular Medicine, University of Copenhagen, Copenhagen, Denmark

**Keywords:** glycosylation, TNFR1, Fas, ST6GAL1, sialic acid, apoptosis, death receptors, cancer

## Abstract

The TNF-TNFR1 signaling pathway plays a pivotal role in regulating the balance between cell survival and cell death. Upon binding to TNF, plasma membrane-localized TNFR1 initiates survival signaling, whereas TNFR1 internalization promotes caspase-mediated apoptosis. We previously reported that the α2-6 sialylation of TNFR1 by the tumor-associated sialyltransferase ST6GAL1 diverts signaling toward survival by inhibiting TNFR1 internalization. In the current investigation, we interrogated the mechanisms underlying sialylation-dependent regulation of TNFR1 and uncovered a novel role for α2-6 sialylation, but not α2-3 sialylation, in mediating apoptosis-resistance. Our studies utilized HEK293 cells with deletion of sialyltransferases that modify *N*-glycans with either α2-3-linked sialic acids (ST3GAL3/4/6) or α2-6-linked sialic acids (ST6GAL1/2). Additionally, ST6GAL1 was re-expressed in cells with ST6GAL1/2 deletion to restore α2-6 sialylation. Using total internal reflection fluorescence (TIRF) microscopy and BS^3^ cross-linking, we determined that, under basal conditions, cells expressing TNFR1 devoid of α2-6 sialylation displayed enhanced TNFR1 oligomerization, an event that poises cells for activation by TNF. Moreover, upon stimulation with TNF, greater internalization of TNFR1 was observed *via* time-lapse TIRF and flow cytometry, and this correlated with increased caspase-dependent apoptosis. These effects were reversed by ST6GAL1 re-expression. Conversely, eliminating α2-3 sialylation did not significantly alter TNFR1 clustering, internalization or apoptosis. We also evaluated the Fas receptor, given its structural similarity to TNFR1. As with TNFR1, α2-6 sialylation had a selective effect in protecting cells against Fas-mediated apoptosis. These results collectively suggest that ST6GAL1 may serve a unique function in shielding cancer cells from apoptotic stimuli within the tumor microenvironment.

Tumor necrosis factor (TNF) is an important pro-inflammatory cytokine and a cornerstone of the TNF superfamily. Since its identification in 1975, TNF has been extensively studied due to its pleiotropic functions including anti-tumor activity (1, 2). TNF serves as a ligand for two receptors, tumor necrosis factor receptor 1 (TNFR1) and tumor necrosis factor receptor 2 (TNFR2). Signaling by TNFR1 can promote either cell survival or cell death, depending upon cell context (3, 4, 5). However, TNFR2 lacks a death domain and cannot initiate apoptosis (6). As another difference, TNFR1 is activated by both soluble and membrane-bound TNF, whereas TNFR2 requires the membrane form of TNF (6). TNFR1 is expressed on nearly all cell surfaces (7). The activation of TNFR1 by TNF contributes to diverse cellular processes spanning cell proliferation, cell differentiation, apoptosis, and cytokine production (1, 8, 9). Dysregulated TNFR1 signaling is a central feature of numerous pathologies, including inflammatory disorders, autoimmune diseases, and cancer (10, 11, 12).

The activation of TNFR1 depends upon the formation of a TNFR1 trimer, followed by higher-order clustering of the trimers (13, 14, 15). It was initially thought that the binding of TNF, itself a trimer (16, 17), was required for TNFR1 trimerization and oligomerization. However, emerging evidence has shown that TNFR1 can oligomerize without TNF stimulation (13, 18, 19). The clustering of TNFR1 in the absence of TNF poises TNFR1 for rapid activation upon cell exposure to TNF. TNFR1 clustering facilitates the TNF-induced internalization of TNFR1 (20, 21), which, in turn, acts as a key determinant in whether activated TNFR1 induces pro-survival or pro-apoptotic signaling cascades. The ability of TNFR1 to instigate either cell survival or cell death depends upon the assembly of distinct intracellular protein complexes following TNF activation (13, 14, 15). The pro-survival arm of the TNFR1 pathway is initiated by TNFR1 localized to the plasma membrane. The binding of TNF to cell surface TNFR1 induces the formation of Complex I, which includes proteins such as TNFR1-associated death domain (TRADD), receptor-interacting serine/threonine-protein kinase 1 (RIPK1), and TNF receptor-associated factor 2 (TRAF2). Complex I promotes the activation of NFκB and subsequent transcription of many pro-survival genes (22). On the other hand, activated TNFR1 oligomers can be internalized to form Complex II *via* the recruitment of caspase 8 and Fas-associated death domain (FADD). Complex II is responsible for inducing caspase-mediated cell death (23). Extensive literature has shown that the internalization of TNFR1 is required for apoptosis. For instance, Schutze’s group used a variety of methods to block TNFR1 internalization and confirmed that retention of TNFR1 on the cell surface prevented caspase activation while concomitantly preserving NFκB-mediated survival signaling (24, 25, 26). Thus, the subcellular localization of TNFR1 is a key factor controlling the toggling between survival and apoptotic signaling.

Given TNFR1’s paramount role in determining cell fate, there is intensive interest in the regulatory mechanisms governing the TNFR1 network. However, the effects of glycosylation on TNFR1 structure and function have received limited attention. Our group previously reported that TNFR1-mediated apoptosis is inhibited by the addition of α2-6-linked sialic acids to the *N*-glycans on TNFR1 (27, 28, 29). The mechanism was found to involve a sialylation-dependent block in TNFR1 internalization (27). Similarly, the α2-6 sialylation of the Fas death receptor (CD95, APO-1) impedes Fas internalization and apoptotic signaling (30). The α2-6 sialic acid linkage on *N*-glycans is directed primarily by the ST6GAL1 sialyltransferase. Another enzyme, ST6GAL2, can also elaborate this linkage; however, ST6GAL2 expression is mostly restricted to the brain (31). The expression of ST6GAL1 is enriched in both stem/progenitor cells and cancer cells when compared with many differentiated epithelial cell types (32, 33, 34). For example, ST6GAL1 is upregulated in pancreatic, colon, ovarian, and gastric cancers, and high ST6GAL1 expression correlates with a poor prognosis (33, 35, 36). We postulate that the ST6GAL1-mediated α2-6 sialylation of TNFR1 and Fas functions as a critical mechanism enabling tumor cell survival within the tumor microenvironment. The ligands for TNFR1 and Fas (TNF and FasL, respectively) are largely produced by immune cells and are prevalent within the inflammatory tumor milieu (37, 38).

While many studies have focused on the general role of sialylation in cancer and other pathologies, there is a dearth of literature addressing the important question of whether the α2-6 and α2-3 sialic acid linkages elicit divergent biological outcomes. The α2-3 sialylation of *N*-glycans is catalyzed by ST3GAL3, ST3GAL4, or ST3GAL6. It is plausible that α2-6 and α2-3 sialylation may differentially affect the structure, and therefore function, of their cognate glycoprotein carriers. Notably, the spatial position of the sialic acid is different for the α2-6 and α2-3 sialic acid linkages to galactose. This could have major structural implications if the *N*-glycan lies within a critical functional domain of a protein. Additionally, the two sialic acid linkages may influence the binding of *N*-glycans to glycan-binding proteins such as galectins (39). Elucidating the biological consequences of α2-6 vs. α2-3 sialylation is essential for understanding how specific sialyltransferases and their distinct sialoglycan products contribute to both organismal homeostasis and pathogenesis.

To interrogate the role of the different sialic acid linkages in TNFR1 signaling, we utilized HEK293 cells engineered with deletions in the genes that add α2-3 sialylation to *N*-glycans (ST3GAL3, ST3GAL4 and ST3GAL6) or in genes that add α2-6 sialylation to *N*-glycans (ST6GAL1 and ST6GAL2). We also re-expressed ST6GAL1 in cells with deletion of endogenous ST6GAL1 and 2 to restore α2-6 sialylation. Using these models, we determined that the α2-6 sialylation of TNFR1 has a selective effect in protecting cells against apoptosis. Specifically, the elimination of α2-6, but not α2-3, sialylation on TNFR1 enhances TNF-induced apoptosis by facilitating TNFR1 oligomerization and internalization. These data suggest that the pervasive upregulation of ST6GAL1 in cancer cells may function to protect cells from the inflammatory tumor microenvironment. Our studies highlight a novel sialylation-dependent TNFR1 regulatory mechanism that controls the switch between cell survival and cell death.

## Results

### HEK293 cell models with differential α2-3 and α2-6 sialylation of N-glycans

**Figure 1:**
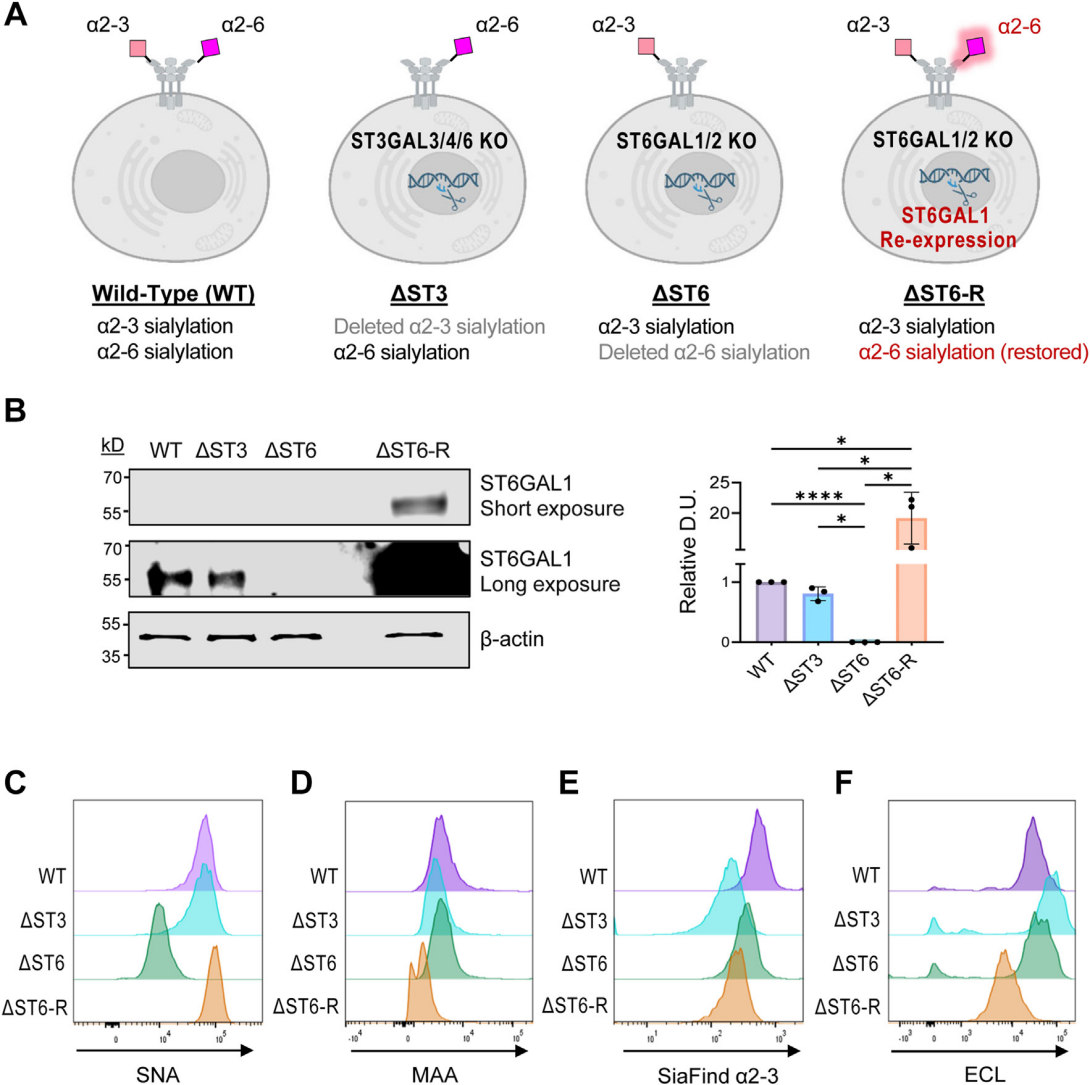
**HEK293 cell models with selective sialyltransferase deletion and restoration.***A*, HEK293 cell lines were engineered with CRISPR/Cas-9-mediated deletion of sialyltransferases involved in the α2-3 or α2-6 sialylation of *N*-glycans. ΔST3 cells (α2-3 deficient) were generated by deletion of ST3GAL3, ST3GAL4, and ST3GAL6. ΔST6 cells (α2-6 deficient) were produced by deleting ST6GAL1 and ST6GAL2. Additionally, we created a stable, polyclonal cell line in which the α2-6 sialylation of *N*-glycans was restored in ΔST6 cells by the re-expression of ST6GAL1 (ΔST6-R). Wild-type (WT) cells represent the unedited HEK293 cell line. *B*, ST6GAL1 expression was evaluated by immunoblotting. Longer exposure of the blot was required to visualize the endogenous ST6GAL1 in WT and ΔST3 cells. The graph shown on the right depicts densitometric analyses of three independent blots for ST6GAL1. Densitometric units (DUs) for ST6GAL1 were normalized to β-actin, and then the relative DU values for the WT line were set at 1.0., with the other cell lines normalized to this value. Results are presented as mean ± S.D., and statistics were calculated using one-way ANOVA with Tukey’s multiple comparison test. (∗*p* < 0.05, ∗∗∗∗*p* < 0.0001). *C*, flow cytometry using SNA lectin confirmed reduced α2-6 sialylation in ΔST6 cells, as well as restoration of α2-6 sialylation in the ΔST6-R line. *D* and *E*, cell surface α2-3 sialylation was assessed by flow cytometry using the MAA lectin (*D*) or SiaFind α2-3 (*E*). *F*, Unsialylated *N*-glycans on the cell surface were evaluated by flow cytometry using ECL lectin.

Wild-type (WT) HEK293 cells produce *N*-glycans with both α2-3 and α2-6 sialylation. HEK293 cells were engineered by CRISPR/Cas9 knockout (KO) of select sialyltransferase genes that direct sialylation of *N*-glycans (Fig. 1*A*) (40). To obtain cells without α2-3 sialylation capacity for *N*-glycans, combinatorial KO of ST3GAL3, ST3GAL4, and ST3GAL6 was used, yielding cells with only α2-6 sialylated *N*-glycans (“ΔST3”). To obtain cells with only α2-3 sialylated *N*-glycans, combinatorial KO of ST6GAL1 and ST6GAL2 was implemented (“ΔST6”). We also generated a cell model in which ST6GAL1 was stably over-expressed in ΔST6 cells to restore α2-6 sialylation (“ΔST6-R”). Re-expression of ST6GAL1 was confirmed by immunoblotting (Fig. 1*B*).

Cell surface sialylation was evaluated by flow cytometry using lectins. Staining with the SNA lectin, which is specific for α2-6 sialic acids, revealed a marked decrease in α2-6 sialylation in the ΔST6 cell line relative to WT and ΔST3 cells (Fig. 1*C*). Surface α2-6 sialylation was restored in the ΔST6-R cells. In fact, levels of α2-6 sialylation in ΔST6-R cells appeared higher than in WT cells, likely because of the more abundant expression of ST6GAL1 in ΔST6-R vs. WT cells (see Fig. 1*B*). Cells were next stained with MAA, which is widely used for the detection of α2-3 sialylation. A reduction in MAA staining was noted in ΔST3 cells as compared with WT cells, although this decrease was relatively small (Fig. 1*D*). MAA recognizes α2-3 sialylation on both *N* and *O*-glycans (41), therefore α2-3 sialylated *O*-glycans would still be detected. As an alternative approach, cells were stained with the SiaFind α2-3 lectin-like protein. This reagent was previously used to verify the loss of α2-3 sialylation in the ΔST3 cell line (42). A much greater reduction in α2-3 sialylation was observed in ΔST3 cells using this reagent (Fig. 1*E*). Interestingly, staining with the MAA and SiaFind α2-3 lectins revealed that α2-3 sialylation was also diminished in the ΔST6-R cell line, although these cells have endogenous expression of ST3GAL3/4/6. We hypothesize that the elevated levels of ST6GAL1 in ΔST6-R vs. WT cells led to more efficient α2-6 sialylation at the expense of α2-3 sialylation due to competition for a subset of *N*-glycan substrates. To evaluate *N*-glycans with uncapped galactoses, we conducted flow cytometry with the ECL lectin, which binds terminal galactose without sialylation. The ΔST3 and ΔST6 cells exhibited a strong increase in unsialylated galactose-containing *N*-glycans, confirming the loss of sialylation. In ΔST6-R cells, ECL staining revealed lower levels of unsialylated galactose compared with WT cells, consistent with the concept that the over-expression of ST6GAL1 resulted in higher levels of terminal sialylation. (Fig. 1*F*).

### Loss of α2-6 sialylation, but not α2-3 sialylation, sensitizes cells to TNFR1-mediated apoptosis

**Figure 2:**
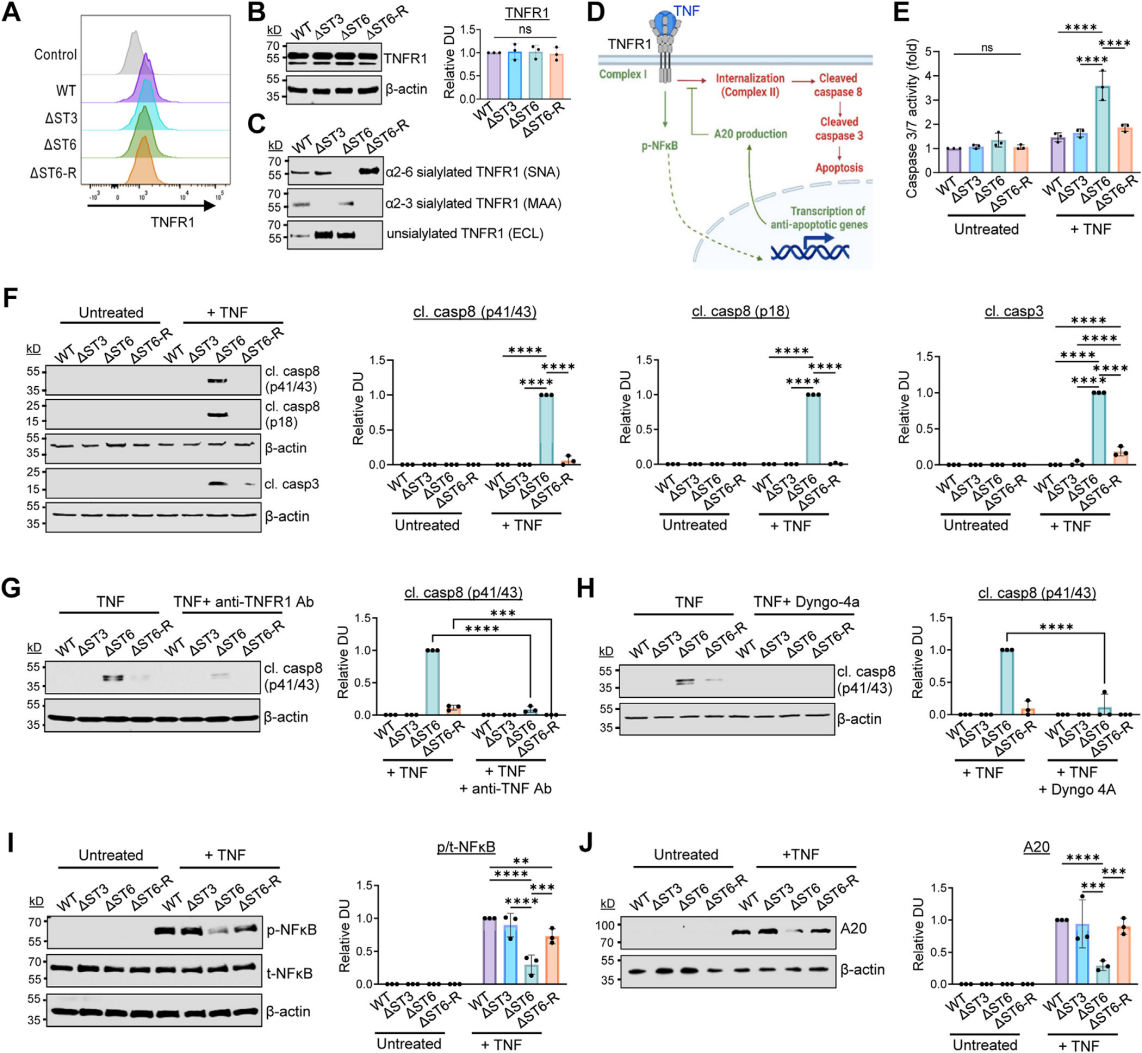
**Deletion of α2-6 sialylation, but not α2-3 sialylation, on TNFR1 enhances TNF-induced apoptosis.***A*, cell surface levels of TNFR1 were analyzed by flow cytometry. *B*, total levels of TNFR1 were evaluated by immunoblotting. Graph on right depicts relative densitometric units (DUs) for TNFR1 normalized to β-actin. The relative DU for WT cells was set at 1.0, and the other lines were normalized to this value. Data were analyzed by one-way ANOVA with Tukey’s test. *C*, Lectin pull-down assays were employed to evaluate the sialylation status of TNFR1. Cell lysates were precipitated with agarose-conjugated SNA, MAA, or ECL lectins, followed by immunoblotting for TNFR1, to determine the amount of α2-6 sialylated, α2-3 sialylated, or unsialylated TNFR1, respectively. *D*, schematic diagram of the TNF-TNFR1 signaling network. Upon stimulation by TNF, surface-localized TNFR1 recruits Complex I, which promotes survival signaling *via* NFκB (*green*). However, internalized TNFR1 induces the formation of Complex II, which drives apoptotic signaling (*red*). *E*, cells were treated with 100 ng/ml TNF for 5 h and assessed for cell death by a caspase 3/7 luminescence assay. Data were analyzed by two-way ANOVA with Tukey’s test. *F*, cells were treated with TNF for 5 h and immunoblotted for cleaved caspase 8 (cl. casp8 p41/43, and p18) and caspase 3 (cl. casp3). Graphs on right depict relative DUs for cleaved caspases 8 and 3 normalized to β-actin. The relative DU for TNF-treated ΔST6 cells was set at 1.0 because there was no detectable signal in either untreated or TNF-treated WT cells. Values for the other cell lines were normalized to the TNF-treated ΔST6 line. Data were analyzed by two-way ANOVA with Tukey’s test. *G*, immunoblotting for cl. casp8 in TNF-treated cells incubated with or without a TNFR1 function-blocking antibody (H398). Graph depicts relative DUs (cl. casp8/β-actin), with values normalized to the TNF-treated ΔST6 line. Statistics were calculated using two-way ANOVA with Tukey’s test. *H*, immunoblotting for cl. casp8 in TNF-treated cells incubated with or without Dyngo-4A, a dynamin inhibitor that blocks TNFR1 internalization. The graph depicts relative DUs (cl. casp8/β-actin), with values normalized to the TNF-treated ΔST6 line. Data were analyzed by two-way ANOVA with Tukey’s test. *I*, cells were treated with or without TNF for 10 min and then lysates were immunoblotted for phosphorylated (p-, pSer536) and total (t-) NFκB p65. Relative DUs were calculated by first normalizing p-NFκB to t-NFκB, and then the p/t NFκB ratio was normalized to β-actin. The relative DU for TNF-treated WT cells was normalized to 1.0. Data were analyzed by two-way ANOVA with Tukey’s test. *J*, cells were treated with or without TNF for 3 h and immunoblotted for A20. The graph depicts relative DUs (A20/β-actin), with values normalized to the TNF-treated WT line. Data were analyzed by two-way ANOVA with Tukey’s test. For all graphs in Figure 2, results are shown as mean ± S.D. from three independent experiments. (nonsignificant (ns) *p* > 0.05, ∗*p* < 0.05, ∗∗*p* < 0.01, ∗∗∗*p* < 0.001, ∗∗∗∗*p* < 0.0001).

The HEK293 cell models were used to interrogate the effects of differential sialylation on TNFR1 signaling. We first confirmed that TNFR1 levels were equivalent in the four cell lines by measuring surface TNFR1 by flow cytometry (Fig. 2*A*), and total TNFR1 expression by immunoblotting (Fig. 2*B*). These data show that modulating sialyltransferase expression did not affect the expression of TNFR1. To examine the sialylation status of TNFR1, we used lectin pull-down assays with MAA, SNA, or ECL, followed by TNFR1 immunoblotting (Fig. 2*C*). In WT cells, TNFR1 displayed both α2-3 and α2-6 sialylation, as well as a low level of unsialylated glycans. In ΔST3 cells, TNFR1 was modified with α2-6, but not α2-3, sialylation, whereas ΔST6 cells expressed TNFR1 with α2-3, but not α2-6, sialylation. Relative to WT cells, ΔST3 and ΔST6 cells had an increased abundance in unsialylated TNFR1. In ΔST6-R cells, TNFR1 was α2-6 sialylated, and the level of α2-6 sialylation was higher than in WT cells, consistent with the over-expression of ST6GAL1 in the ΔST6-R line. However, α2-3 sialylation of TNFR1 was reduced in ΔST6-R cells, which may be due to competition between ST6GAL1 and α2-3 sialyltransferases, as posited above. The level of unsialylated TNFR1 was lower in ΔST6-R cells as compared with WT cells, suggesting that the over-expression of ST6GAL1 led to more extensive capping of terminal galactoses.

In the canonical TNFR1 pathway (Fig. 2*D*), the binding of TNF to plasma membrane-localized TNFR1 induces the formation of Complex I and activation of NFκB-mediated survival signaling. However, after initial activation by TNF, TNFR1 may be internalized, which instigates the recruitment of Complex II and the activation of caspase-mediated cell death. To monitor apoptosis, HEK293 cells were treated with or without TNF for 5 h and subjected to activity assays for the executioner caspases, caspase 3 and 7. Fig. 2*E* shows significantly increased TNF-induced caspase activation in ΔST6 cells compared with WT and ΔST3 cells. Importantly, this enrichment in caspase activation was reversed in ΔST6-R cells, indicating that the re-expression of ST6GAL1 prevented apoptosis. Apoptosis was also evaluated by immunoblotting for cleaved forms of caspases 8 and 3. Caspase 8 is an initiator caspase specific to the extrinsic apoptotic pathway activated by death receptors including TNFR1 and Fas. Enhanced cleavage of caspases 8 and 3 was noted in ΔST6 cells at 5 h following TNF treatment, and this was reversed in the ΔST6-R cells (Fig. 2*F*).

To confirm that TNF-induced apoptosis was due to signaling by TNFR1, cells were pre-treated with a function-blocking antibody against TNFR1. As shown in Figure 2*G*, the TNFR1 blocking antibody attenuated TNF-induced caspase activation. We also verified that TNFR1 internalization was required for caspase activation, as reported (17, 19, 33). Pre-treatment of cells with Dyngo-4a, a dynamin inhibitor that prevents receptor endocytosis, eliminated caspase activation induced by TNF (Fig. 2*H*).

Our previous studies indicated that the α2-6 sialylation of TNFR1 shunts downstream signaling toward survival (27). Hence, we examined the survival arm of the TNFR1 pathway by immunoblotting for phosphorylated (activated) NFκB p65 (Fig. 2*I*). After 10 min of TNF treatment, WT and ΔST3 cells had much greater activation of NFκB than ΔST6 cells. However, compared with ΔST6 cells, ΔST6-R cells had increased levels of p-NFκB, indicating a restoration of survival signaling. NFκB acts as a key transcription factor downstream of TNFR1 to stimulate the expression of anti-apoptotic molecules including A20 (TNFAIP3). At 3 h following TNF treatment, an increase in A20 was observed in all cell lines; however, ΔST6 cells had markedly lower levels of A20 as compared with WT, ΔST3, and ΔST6-R cells (Fig. 2*J*). The combined results in Figure 2 show that the loss of α2-6 sialylation, but not α2-3 sialylation, on TNFR1, enhances TNF-induced apoptosis while simultaneously inhibiting pro-survival signaling. It is also noteworthy that the behavior of ΔST6-R cells, with primarily α2-6 sialylated TNFR1, phenocopies that of WT cells with both α2-6 and α2-3 sialylated TNFR1, further suggesting a dominant role for α2-6 sialylation in regulating TNFR1-induced apoptosis.

### Increased ligand-independent clustering of TNFR1 is observed in cells lacking α2-6 sialylated TNFR1

**Figure 3:**
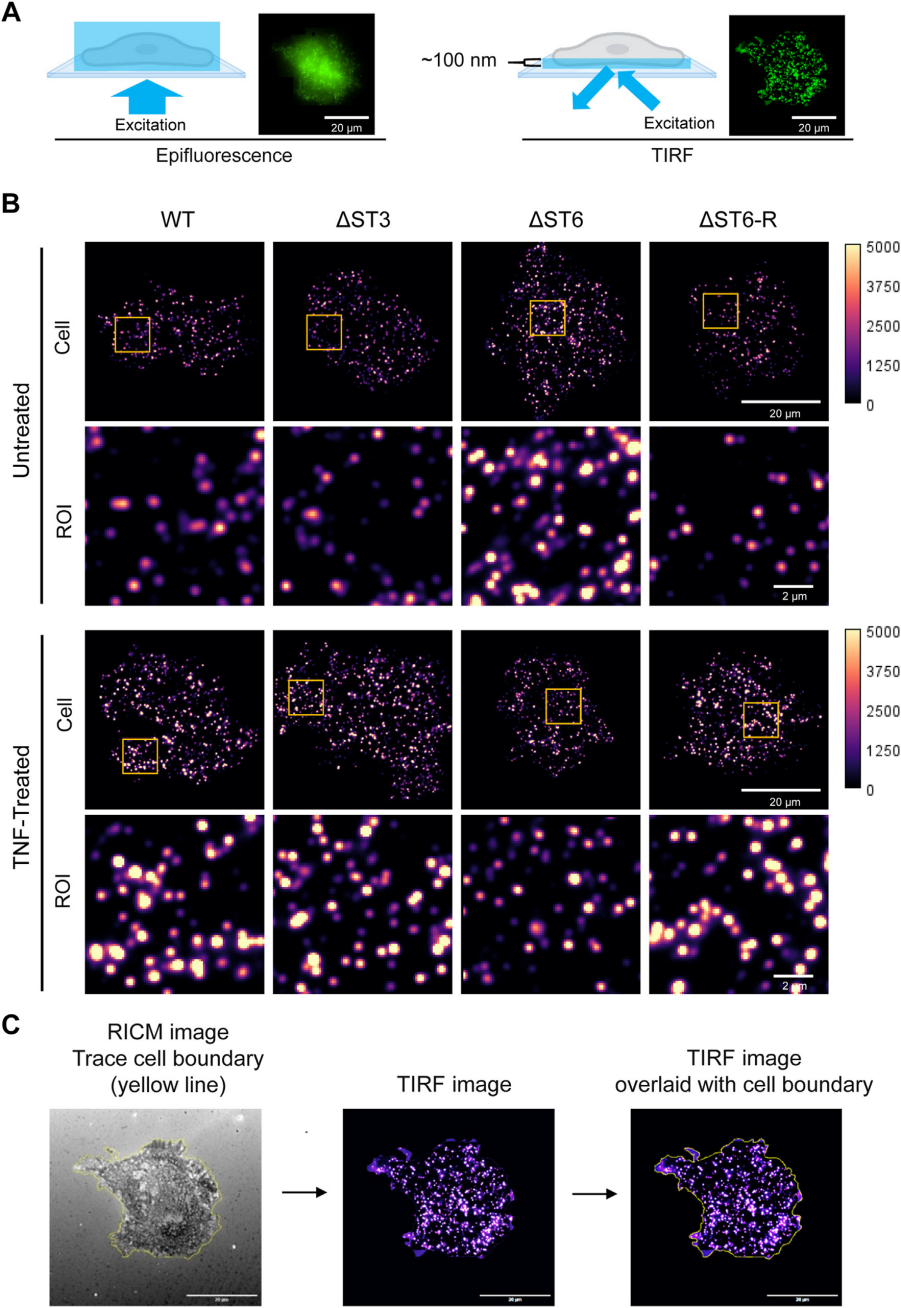
**Evaluation of TNFR1 clustering by TIRF microscopy.***A*, schematic diagram illustrating the difference between epifluorescence and TIRF microscopy. Epifluorescence imaging produces an out-of-focus image with a diffused TNFR1 protein signal. TIRF illumination allows selective acquisition of a higher signal-to-noise image focused within a ∼100 nm region at the plasma membrane-coverslip interface. Scale bar represents 20 μm. *B*, TIRF microscopy was performed on untreated cells or cells treated with 100 ng/ml TNF for 15 min. Cells were fixed and stained with an anti-TNFR1 antibody to visualize TNFR1 clusters. Each image depicts TNFR1 clusters within a single cell. Scale bars represent 20 μm for the field of view and 2 μm for the region of interest (ROI). *C*, RICM was used to define the cell boundary (*yellow line*), and this boundary was overlaid onto the TIRF image to measure TIRF signals relative to the cell area.

**Figure 4:**
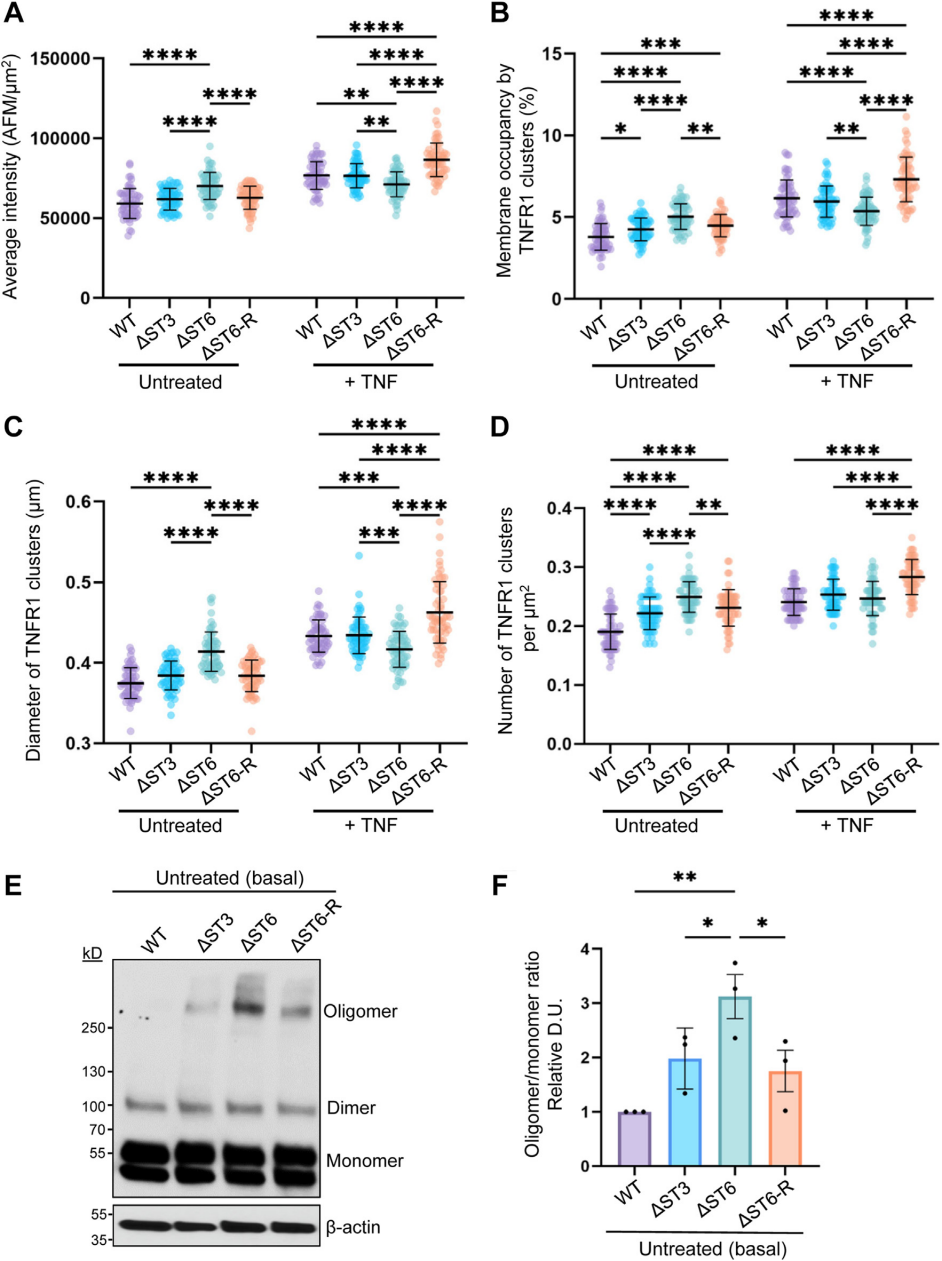
**Cells lacking α2-6 sialylated TNFR1 exhibit increased TNFR1 oligomerization in the absence of ligand.** Cells treated with or without TNF for 15 min were stained with an anti-TNFR1 antibody and then imaged using TIRF microscopy to detect TNFR1-positive punctae, representing TNFR1 clusters. *A*, the average intensity per image (arbitrary fluorescence units per μm^2^, AFM/μm^2^). *B*, the percentage of the basal cell membrane occupied by TNFR1 clusters. *C*, the average diameter (μm) of TNFR1 clusters per image. *D*, the number of TNFR1 clusters per μm^2^. Graphs in panels (*A*–*D*) depict mean ± S.D. from two independent experiments with 60 cells analyzed per group with statistical analysis by two-way ANOVA with Tukey’s test (∗*p* < 0.05, ∗∗*p* < 0.01, ∗∗∗*p* < 0.001, ∗∗∗∗*p* < 0.0001). *E*, cross-linking of receptors using BS^3^, followed by TNFR1 immunoblotting, was conducted on untreated cells to evaluate the formation of TNFR1 oligomers in the absence of TNF. *F*, TNFR1 oligomers (>250 kDa) and monomers were quantified by densitometry, and data were plotted as the ratio of oligomers to monomers. Values for the WT line were set at 1.0, and the other cell lines were normalized to this value. Graph depicts mean ± S.D. from three independent experiments. Data were analyzed using a one-way ANOVA with Tukey’s test (∗*p* < 0.05, ∗∗*p* < 0.01).

The clustering and subsequent internalization of TNFR1 are pivotal steps in TNFR1-induced apoptosis (20). To examine TNFR1 clustering, we used total internal reflection fluorescence (TIRF) microscopy, which selectively illuminates a ∼100 nm region encompassing the basal plasma membrane adjacent to the coverslip (Fig. 3*A*). Compared with the diffuse signal yielded by standard epifluorescence microscopy, the selective excitation employed by TIRF microscopy allows for the specific identification and quantification of plasma membrane associated-proteins (43). Cells were treated with or without TNF for 15 min, and then fixed and stained for TNFR1. Representative TIRF images reveal TNFR1 clusters at the plasma membrane (Fig. 3*B*). The white/yellow hue indicates higher fluorescence intensity, which correlates with a high density of TNFR1 molecules within the cluster, and magenta/purple has a lower intensity, as indicated by the calibration bar. Reflection interference contrast microscopy (RICM) was used to define the cell boundary, which was utilized to obtain a measurement of the cell area (Fig. 3*C*). In untreated cells, the average TNFR1 fluorescence intensity was higher in ΔST6 cells compared with WT, ΔST3, and ΔST6-R cells (Fig. 4*A*). However, following TNF treatment, ΔST6 cells displayed a decrease in TNFR1 intensity relative to the other cell lines.

Because of the importance of TNFR1 clustering in receptor internalization and downstream signaling, we examined clusters in several ways. We first examined the overall percentage of the cell membrane occupied by TNFR1 clusters. In the absence of TNF, ΔST6 cells had a greater percentage of TNFR1 membrane occupancy compared with the other cell lines (Fig. 4*B*). After TNF stimulation, the amount of membrane occupied by TNFR1 clusters was lower in ΔST6 cells than in WT, ΔST3, and ΔST6-R cells.

We next quantified the average diameter of TNFR1 clusters (Fig. 4*C*). Under basal conditions, ΔST6 cells had significantly larger TNFR1 clusters compared with WT, ΔST3, and ΔST6-R cells. However, smaller clusters were observed in ΔST6 cells after TNF treatment relative to the other cell lines.

Finally, the number of TNFR1 clusters was enriched in the ΔST6 cell line under basal conditions (Fig. 4*D*). For TNF-treated cells, the cluster number was comparable in WT, ΔST3, and ΔST6 cells, whereas ΔST6-R cells had more clusters. Statistical analyses comparing untreated vs. TNF-treated cells revealed that all of the cell lines except ΔST6 responded to TNF with an increase in the TNFR1 clustering parameters (Fig. S1). The results in Figure 4, *A*–*D* suggest that the loss of α2-6 sialylation on TNFR1 had a pronounced effect on the clustering of TNFR1 in the absence of ligand. We hypothesize that the increased clustering of TNFR1 in ΔST6 cells under basal conditions poises the cells for more robust apoptotic signaling upon exposure to TNF. Following TNF treatment, clustering parameters were reduced in ΔST6 cells, which could be due to enhanced TNF-induced TNFR1 internalization.

### Receptor cross-linking experiments confirm enhanced basal clustering of TNFR1 devoid of α2-6 sialylation

To confirm the finding that loss of α2-6 sialylation facilitated ligand-independent TNFR1 clustering, we treated cells under basal conditions with Bis(sulfosuccinimidyl) suberate (BS^3^), a cross-linking reagent commonly used to study TNFR1 oligomerization (44, 45). Cells were treated with BS^3^ for 30 min on ice (to prevent TNFR1 internalization), followed by cell lysis and immunoblotting for TNFR1. Levels of monomeric and dimeric TNFR1 were comparable among the four cell lines; however, there was a substantial increase in the abundance of higher-order oligomers (>250 kDa) in ΔST6 cells relative to WT and ΔST3 cells (Fig. 4*E*). Importantly, ΔST6-R cells had lower levels of TNFR1 oligomers than ΔST6 cells, consistent with the restoration of α2-6 sialylation. Densitometric quantification of the oligomer-to-monomer ratio confirmed greater oligomer formation in ΔST6 cells (Fig. 4*F*), which was attenuated in the ΔST6-R cells. There was also a trend toward increased oligomerization in the ΔST3 cells, however these results were not statistically significant. Taken together, data in Figure 4 suggest that the presence of α2-6 sialylation on TNFR1 hinders the formation of higher-order oligomers.

### Live cell TIRF microscopy reveals more rapid TNF-induced internalization of TNFR1 lacking α2-6 sialylation

The TIRF results in Figure 4 revealed that, in addition to basal TNFR1 oligomerization, TNF-induced TNFR1 clustering was differentially affected by the α2-3 vs. α2-6 sialic acid linkages. Compared with WT, ΔST3 and ΔST6-R cells, ΔST6 cells had a lower abundance of TNF-induced surface TNFR1 oligomers as indicated by a reduction in the average intensity, the percentage of membrane area occupied by TNFR1 clusters, and the diameter of clusters. We postulated that TNF stimulated more robust internalization of TNFR1 clusters in the ΔST6 cell line. To address this hypothesis, we conducted live-cell TIRF microscopy on cells transfected with a TNFR1 construct tagged with GFP at the C-terminus (TNFR1-GFP). This approach enabled an assessment of dynamic changes in TNFR1 within the plasma membrane, including receptor internalization. Cells expressing TNFR1-GFP were imaged before (basal) and after TNF treatment, with images captured every 2 min for 30 min. The extended data depicting TIRF images at 2-min intervals are presented in Fig. S2.

**Figure 5:**
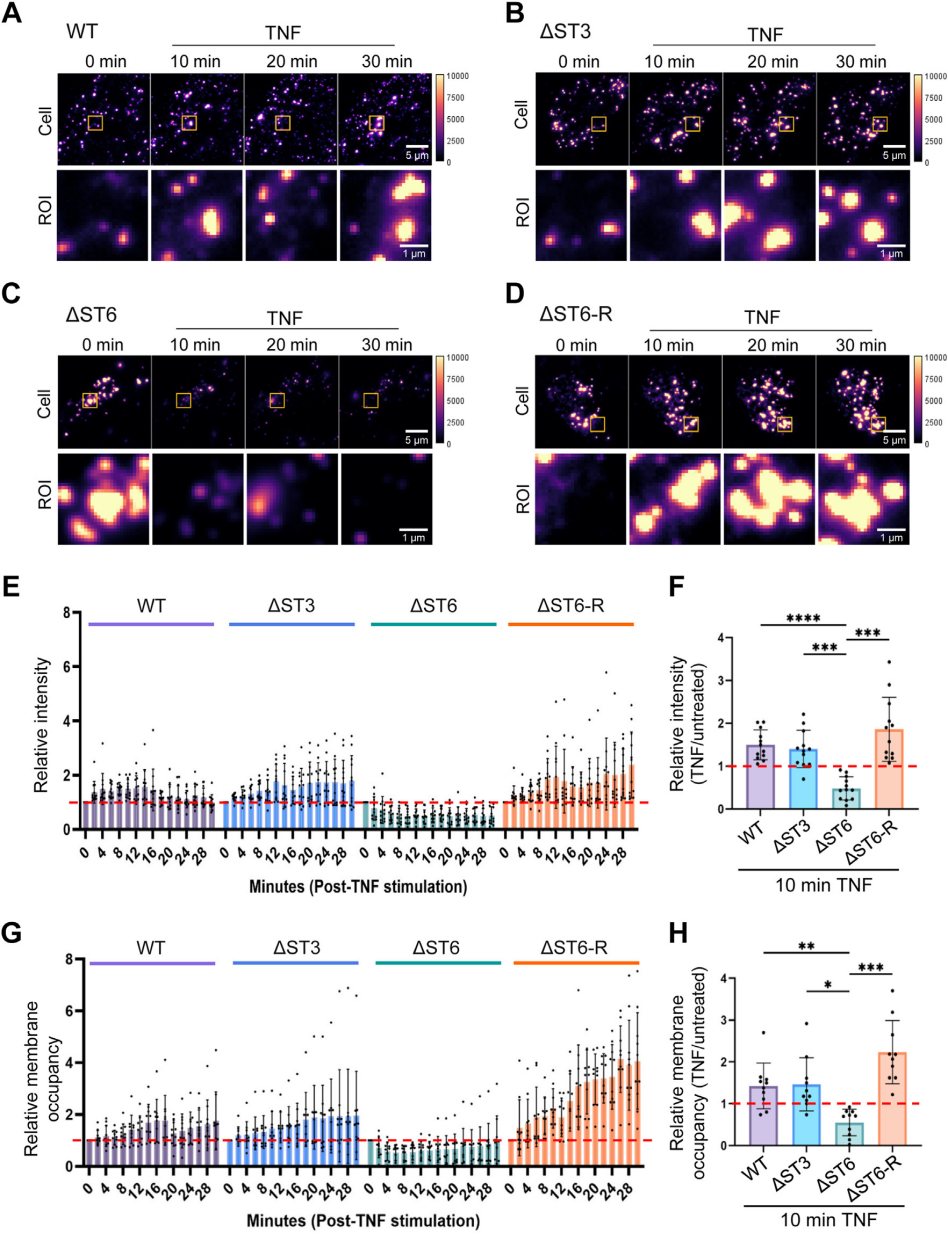
**Live cell TIRF reveals that TNFR1 lacking α2-6 sialylation is more rapidly internalized upon TNF stimulation.** Time-lapse TIRF imaging of live cells transfected with TNFR1-GFP was performed to evaluate TNFR1 dynamics. Images were acquired every 2 min beginning immediately before TNF treatment (0 min) and extending for 30 min afterward. *A*–*D*, representative images of WT (*A*), ΔST3 (*B*), ΔST6 (*C*), and ΔST6-R (*D*), cells before and 10, 20, and 30 min after TNF treatment. Each individual image depicts TNFR1 clusters within a single cell. Scale bar for the field of view = 5 μm, region of interest (ROI) = 1 μm. *E*, the average fluorescence intensity of TIRF images was quantified and normalized to the average fluorescence intensity of the time 0/untreated cells (*dashed red line*) to enable a direct comparison of TNF-induced changes in intensity. *F*, graph depicts the fold differences in relative intensity for cells treated with TNF for 10 min as compared with untreated cells (*dashed red line*). Values represent mean ± S.D. from two independent experiments using 12 cells analyzed per group. Statistical analyses were conducted using one-way ANOVA with Tukey’s test (∗∗∗*p* < 0.001, ∗∗∗∗*p* < 0.0001). *G*, the proportion of the plasma membrane occupied with TNFR1-GFP clusters was measured. Values for TNF-treated cells were normalized to time 0/untreated cells (dashed red line). *H*, graph depicts the fold differences in the proportion of membrane occupied by TNFR1-GFP clusters in cells treated with TNF for 10 min as compared with untreated cells (dashed red line). Values represent mean ± S.D. from two independent experiments using 10 cells analyzed per group. Statistical analyses were performed using one-way ANOVA with Tukey’s test (∗*p* < 0.05, ∗∗*p* < 0.01, ∗∗∗*p* < 0.001).

Representative TIRF images are shown for cells immediately before TNF treatment (0 min) and then at 10-min intervals after treatment (Fig. 5, *A*–*D*). As illustrated in the ROIs, TNFR1 clusters appeared and disappeared over the course of TNF treatment. While there were no obvious distinctions in the WT, ΔST3, and ΔST6-R cells (Fig. 5, *A*, *B* and *D*, respectively), a stark difference was apparent in the ΔST6 cell line (Fig. 5*C*). At 10 min after TNF treatment, most of the TNFR1 clusters had disappeared from the surface of ΔST6 cells, and the level of surface clusters remained low throughout the 30-min experiment.

We quantified the amount of TNFR1-GFP on the plasma membrane at each timepoint. The average intensity was normalized to the untreated cells (0 min) to allow a direct comparison of the TNF-induced changes across the four cell lines. TNF treatment stimulated an increase in the intensity of TNFR1-GFP in WT, ΔST3, and ΔST6-R cells (Fig. 5*E*). In striking contrast, the TNFR1-GFP intensity was reduced in ΔST6 cells as early as 2 min after TNF treatment, and values remained suppressed for the duration of the 30-min treatment. We conducted a statistical analysis of the relative intensity for the four cell lines across all time points (Fig. S3 and Table S1). As a representative example, the relative intensity at 10 min is presented in Figure 5*F*. For WT, ΔST3, and ΔST6-R, TNF treatment stimulated an increase in TNFR1-GFP intensity relative to basal conditions (dashed red line), indicating an increase in plasma membrane-localized TNFR1-GFP clusters. In contrast, ΔST6 cells had reduced TNFR1-GFP intensity, consistent with a loss in surface clusters.

We next calculated the proportion of the cell membrane occupied by TNFR1-GFP clusters. In WT, ΔST3, and ΔST6-R cells, the proportion of membrane occupancy by TNFR1-GFP clusters following TNF treatment increased, whereas membrane occupancy was decreased in ΔST6 cells (Figs. 5*G*, S4 and Table S2). The 10-min time point for TNF treatment is shown in Figure 5*H*. These results suggest that there is more rapid TNF-induced internalization of TNFR1 in cells lacking α2-6-sialylated TNFR1.

### More efficient TNF-induced internalization of TNFR1 is observed in the absence of α2-6 sialylation

**Figure 6:**
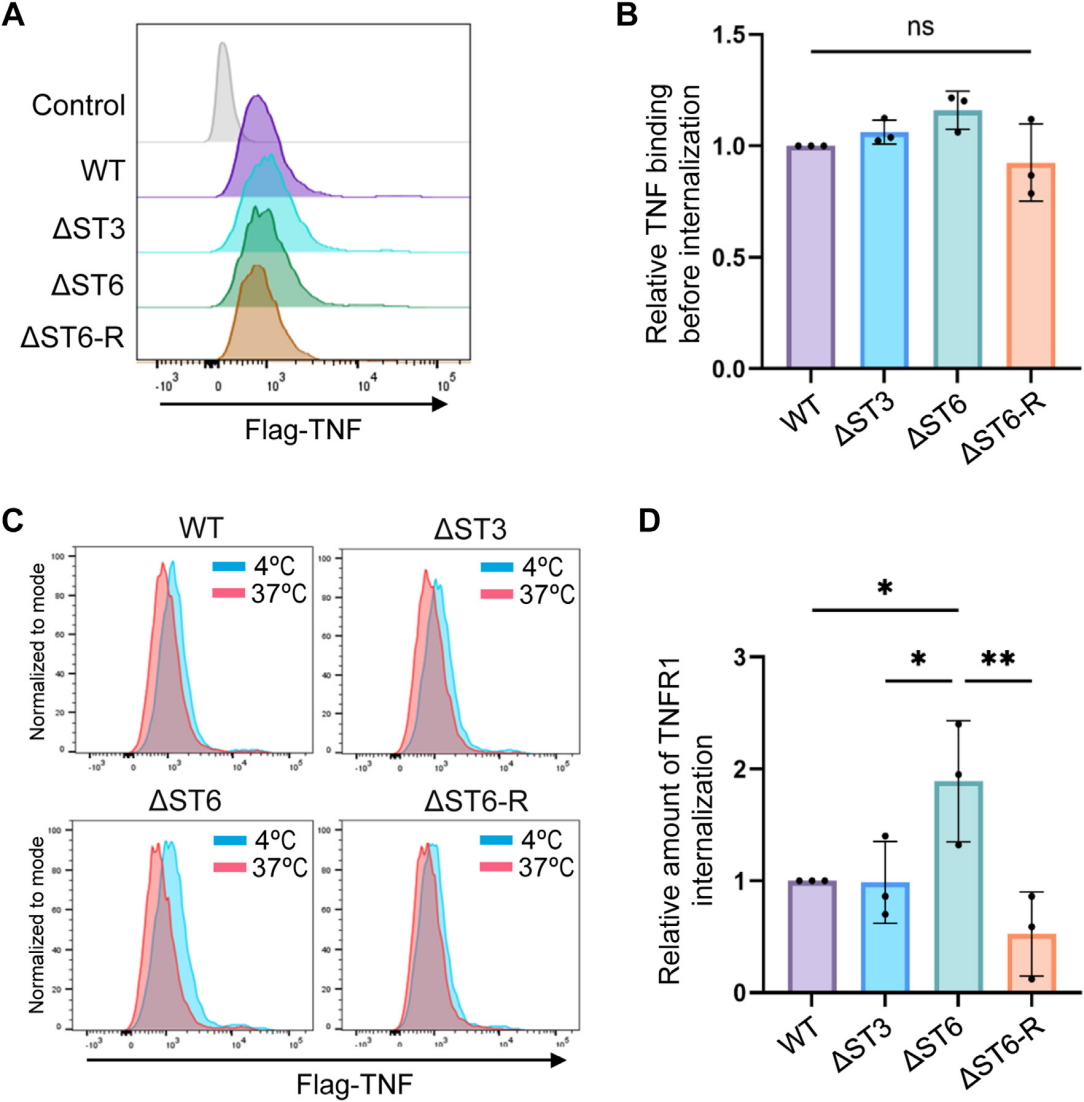
**The loss of α2-6 sialylation on TNFR1 promotes enhanced TNF-induced TNFR1 internalization as measured by flow cytometry.***A*, cells were treated with Flag-tagged TNF on ice for 60 min, which allows the binding of TNF to TNFR1 but does not permit the internalization of TNF-TNFR1 complexes. The amount of TNF bound to the cell surface at the end of the 60-min binding interval was measured by flow cytometry using an antibody against the Flag tag. A representative experiment is shown. *B*, TNF binding to the cell surface was quantified by measuring MFI, and MFI values were then normalized to the WT cell line. The graph depicts mean ± S.D. from three independent experiments with statistical analysis by one-way ANOVA with Tukey’s test (ns: *p* > 0.05). *C*, following the 60-min TNF binding interval, cells were switched to 37 °C for 30 min to allow the internalization of TNF-TNFR1 complexes. The TNF-TNFR1 complexes remaining on the cell surface after the temperature switch were measured by staining cells with the anti-Flag antibody. Representative histograms depict the level of surface TNF-TNFR1 complexes before (4 °C) and after (37 °C) the internalization step. *D*, the MFI values for surface TNF-TNFR1 complexes before and after the internalization step were compared to obtain a measurement of the degree of TNFR1 internalization. Values for internalization were normalized to the WT cell line. The graph depicts mean ± S.D. from three independent experiments with statistical analysis by one-way ANOVA with Tukey’s test (∗*p* < 0.05, ∗∗*p* < 0.01).

TNFR1 internalization was further evaluated using a flow cytometric method employed previously to study TNFR1 surface dynamics (27, 46). Cells were incubated with Flag-tagged TNF for 60 min on ice, which allows TNF binding to TNFR1, but prevents the internalization of TNF-TNFR complexes. The amount of TNF bound after the 60-min interval on ice was quantified by staining cells with an anti-Flag antibody (representative experiment in Figure 6*A*). There was no significant difference in the amount of TNF bound to the four different cell lines (Fig. 6*B*). TNFR1 internalization was then examined by switching cells with bound TNF to 37 °C for 30 min. After this interval, cells were stained with the anti-Flag antibody to assess the levels of TNF-TNFR1 complexes remaining on the cell surface after the internalization step. Figure 6*C* shows a representative experiment comparing surface TNF-TNFR1 complexes before (4 °C) and after (37 °C) internalization. The relative amount of internalization was calculated by comparing the level of surface TNF-TNFR1 complexes before and after the internalization step. Significantly increased internalization of TNFR1 was observed in ΔST6 cells compared with WT and ΔST3 cells, and this effect was reversed in the ΔST6-R cells (Fig. 6*D*).

### Loss of α2-6 sialylation on the Fas receptor promotes Fas-mediated apoptosis

**Figure 7:**
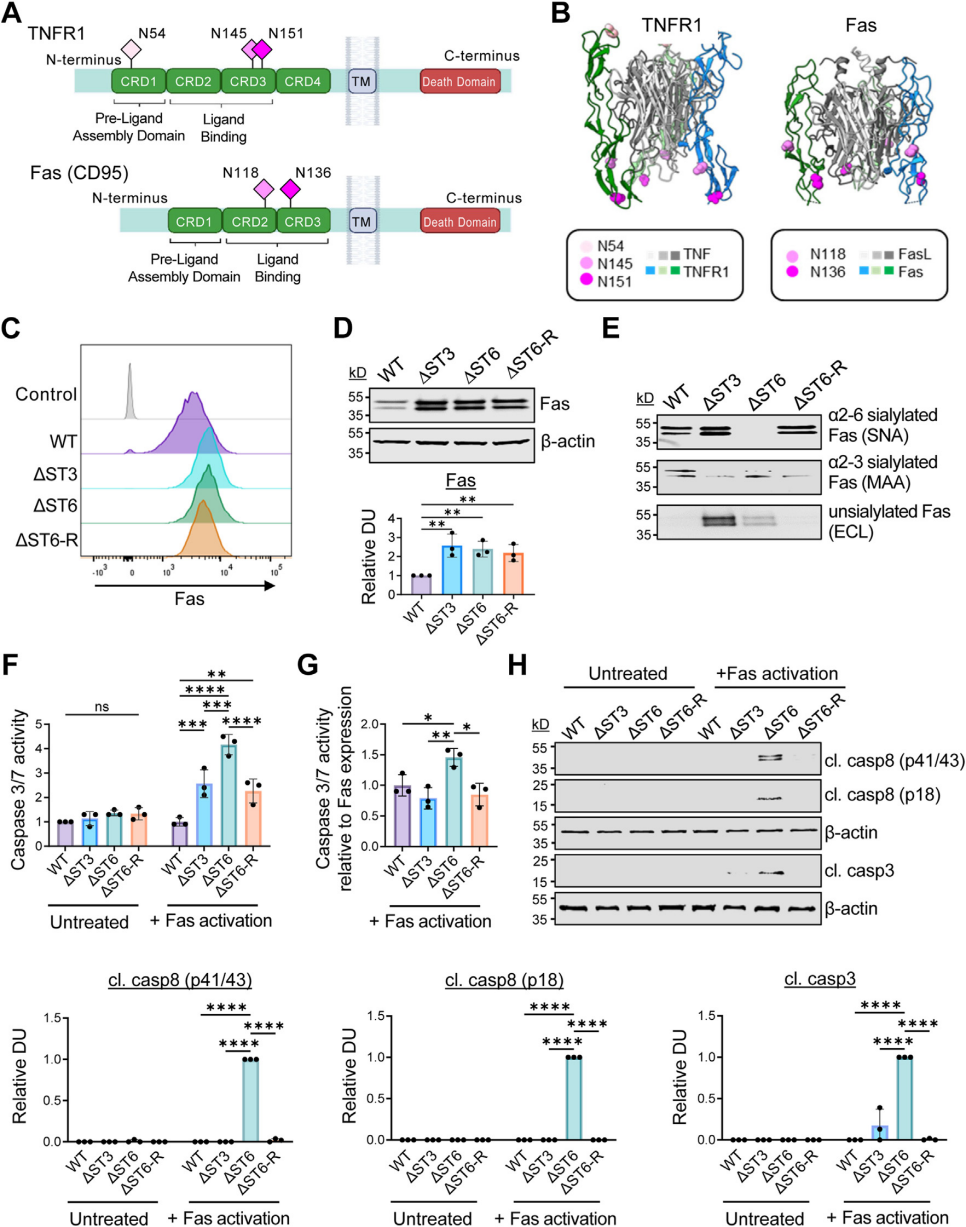
**The loss of α2-6 sialylation on the Fas death receptor enhances Fas-mediated apoptosis.***A*, schematic diagrams of the human TNFR1 and Fas structures. Potential sites for the addition of sialylated *N*-glycans are indicated by pink diamonds. CRD: Cysteine rich domain; TM: transmembrane helix. *B*, molecular models from the crystal structures of the extracellular domains are shown for: the TNF-TNFR1 complex (PDB: 7KP7, note that this structure lacks CRD4); and the FasL-Fas complex (homology model based on PDB 7KP7, 5L19, 3TJE). The individual monomers within the ligand trimers (TNF and FasL) are indicated by varying shades of *grey*, and the monomers within the receptor trimers (TNFR1 and Fas) are shown in shades of *blue* and *green*. Sites for *N*-glycosylation are indicated in *pink*. *C*, flow cytometry was performed to assess the levels of Fas on the cell surface. *D*, total Fas expression was evaluated by immunoblotting. The graph depicts relative DUs (Fas/β-actin), with values for the WT line normalized to 1.0. Data were analyzed by one-way ANOVA with Tukey’s test. *E*, cell lysates were precipitated with agarose-conjugated SNA, MAA, or ECL lectin and immunoblotted for Fas to determine the amount of α2-6, α2-3 sialylated, or unsialylated Fas, respectively. *F*, cells were treated for 5 h in the presence or absence of the Fas agonistic antibody, CH11. Fas-mediated apoptosis was assessed by a caspase 3/7 luminescence assay. Data were analyzed using two-way ANOVA with Tukey’s test. *G*, results from the caspase 3/7 activity assays in panel F were normalized to the level of Fas expression, as measured by densitometric analyses of Fas immunoblots in panel *D*. Data were analyzed using one-way ANOVA with Tukey’s test. *H*, lysates from cells treated with or without the agonistic CH11 antibody were immunoblotted for cl. casp8 (p41/43, and p18) and cl. casp3. Graphs depict relative DUs for cl. casp8 or cl. casp3 normalized to β-actin. Relative DU for the CH11-treated ΔST6 line was normalized to 1.0 because there was no detectable signal in either untreated or CH11-treated WT cells. Data were analyzed using two-way ANOVA with Tukey’s test. For all graphs in Figure 7, data are presented as mean ± S.D. from three independent experiments. (∗*p* < 0.05, ∗∗*p* < 0.01, ∗∗∗*p* < 0.001, ∗∗∗∗*p* < 0.0001).

Our prior work showed that, like TNFR1, α2-6 sialylation of the Fas death receptor impedes Fas internalization and subsequent activation of caspases (30). TNFR1 and Fas have very similar structures, consisting of an extracellular domain, a transmembrane domain, and an intracellular domain containing the death domain, which activates the apoptotic cascade (Fig. 7*A*). The extracellular domain of TNFR1 includes four cysteine-rich domains (CRDs) while Fas has three CRDs. CRD1 contains the pre-ligand assembly domain (PLAD), which promotes the formation of TNFR1 and Fas multimers in the absence of ligand, and also contributes to ligand-dependent oligomerization (21, 47). Human TNFR1 has three Asn residues with consensus sequences for *N*-glycosylation (N x S/T, where x is any amino acid except Pro). These sites are N54, N145, and N151 (the numbering scheme includes the signal sequence). N54 is located in the PLAD region of CRD1; whereas N145 and N151 are in CRD3. The human Fas receptor has two *N*-glycosylation sites, N118 in CRD2 and N136 in CRD3. Molecular models from the crystal structures of human TNFR1 and Fas show that some of the *N*-glycosylation sites on these two receptors have very similar positions, for example, N145 on TNFR1 and N118 on Fas, and N151 on TNFR1 and N136 on Fas (Fig. 7*B*).

In light of the comparable structures of TNFR1 and Fas, combined with the similar positioning of the *N*-glycans, we tested the effects of the different sialic acid linkages on Fas-mediated apoptosis. Surface levels of Fas were evaluated by flow cytometry and total Fas expression was measured by immunoblotting (Fig. 7, *C* and *D*, respectively). Compared with WT cells, elevated Fas expression was noted in the ΔST3, ΔST6, and ΔST6-R cell lines. However, there was no difference in Fas expression between the ΔST3, ΔST6, and ΔST6-R models. Two bands for Fas were observed, as reported by others (48). The sialylation status of Fas was assessed using lectin pull-down assays (Fig. 7*E*). WT cells expressed both α2-3 and α2-6 sialylated Fas, while unsialylated forms of Fas were not detected. ΔST3 cells had strong expression of α2-6 sialylated Fas, and a negligible level of α2-3 sialylated Fas, as expected. Interestingly, Fas retained some detectable α2-3 sialylation in ΔST3 cells, in contrast to TNFR1 (see Fig. 2*C*). The reason for this discrepancy is unclear, however, it is worth mentioning that Fas is reportedly modified by *O*-glycosylation (49). It is possible that the MAA pull-down experiments precipitated a small amount of Fas with α2-3 sialylated *O*-glycans. In line with the reduced α2-3 sialylation noted in ΔST3 cells, unsialylated Fas glycoforms were greatly increased relative to WT cells. In ΔST6 cells, Fas was modified with α2-3, but not α2-6, sialylation and an increase in unsialylated Fas was apparent as compared with WT cells. In the ΔST6-R cell line, Fas had high levels of α2-6 sialylation, low levels of α2-3 sialylation, and an undetectable level of unsialylated glycans.

Cells were treated with the Fas-activating antibody, CH11, and monitored for apoptosis. Activity assays for caspases 3 and 7 revealed that ΔST6 cells had greater caspase activation than ΔST3 cells, and this enhancement in apoptosis was reversed in ΔST6-R cells (Fig. 7*F*). Of note, ΔST3 and ΔST6-R cells had increased ligand-induced caspase activation compared with WT cells, which contrasts with the equivalent levels of TNF-induced apoptosis in WT, ΔST3, and ΔST6-R cells (see Fig. 2*E*). This is likely due to the fact that ΔST3 and ΔST6-R cells have higher expression of Fas than WT cells. To address this issue, we used densitometry to quantify Fas on immunoblots and then normalized the amount of caspase 3/7 activity to the level of Fas expression. Figure 7*G* shows that, when normalized to Fas expression, ligand-induced caspase activation was significantly higher in ΔST6 cells, but not in ΔST3 or ST6-R cells, relative to WT cells. In agreement with caspase activity assays, ΔST6 cells exhibited increased cleavage of caspases 8 and 3 as compared with the other cell lines (Fig. 7*H*). In the aggregate, these results suggest that the α2-6 sialylation, but not α2-3 sialylation, of Fas and TNFR1 has a protective effect against apoptosis.

## Discussion

A disruption in TNF-TNFR1 signaling contributes to the pathogenesis of multiple diseases including autoimmune disorders and cancer (10, 11, 12). Although TNF was initially named for its tumoricidal activity (50), later studies aimed at exploiting TNF to treat cancer revealed that the TNF-TNFR1 axis can also promote cell survival and proliferation *via* activation of NFκB (51). This duality in TNF-TNFR1 signaling poses challenges with regard to the therapeutic targeting of this pathway. An in-depth understanding of the molecular mechanisms regulating TNFR1 structure and function is urgently needed to develop more effective clinical treatments.

A comprehensive literature has established a role for subcellular localization of TNFR1 in directing a cell’s response to TNF (3, 4, 5). For instance, caspase activation, but not NFκB activation, is blocked by interventions that prevent TNFR1 internalization, including treatment with a dynamin inhibitor, deletion of the TNFR1 internalization domain, and transfection with Adenovirus protein 14.7K (24, 26, 52). As with TNFR1, apoptotic signaling by the Fas death receptor is dependent upon ligand-induced Fas internalization (53, 54). Our prior studies showed that the internalization of TNFR1 and Fas, and consequent induction of apoptosis, were inhibited by ST6GAL1-mediated α2-6 sialylation (27, 28, 29, 30). In the current investigation, we used HEK293 cells engineered with genetic deletion of the sialyltransferases that cap *N*-glycans with either α2-3 or α2-6 sialic acids to delve more deeply into sialylation-dependent mechanisms that regulate TNFR1 and Fas. Results herein suggest that the inhibitory effect of α2-6 sialylation on receptor internalization may be secondary to alterations in receptor clustering. TNFR1 clustering is vital for the initiation and amplification of downstream signaling (20, 21). In the absence of α2-6 sialylation, TNFR1 exhibited increased ligand-independent clustering, a process known to poise cells for TNF stimulation. Upon cell exposure to TNF, clusters composed of TNFR1 receptors lacking α2-6 sialylation were more rapidly and efficiently internalized. Correspondingly, cells devoid of α2-6 sialylation had enhanced caspase activation, but reduced activation of NFκB. All of these effects were reversed by the re-expression of ST6GAL1, which aligns with our previous studies indicating that ST6GAL1 activity diverts TNFR signaling toward survival (27). Importantly, the loss of α2-3 sialylation on TNFR1 *N*-glycans did not affect the clustering or internalization of TNFR1, nor did it alter NFκB or caspase activation.

The role of variant sialylation in regulating the structure of TNFR1 and Fas remains to be determined. However, it is worth noting that the 3D structural models for TNFR1 and Fas suggest that some of the Asn residues with consensus sequences for *N*-glycosylation have comparable positions within the two receptors (see Fig. 7*B*). More specifically, there is a similarity in the positioning of N145 on TNFR1 and N118 on Fas, as well as N151 on TNFR1 and N136 on Fas. Intriguingly, these four sites lie within, or near, an important domain found in all death receptors known as the “90s” loop, which ranges from ∼10-15 amino acids (55). The 90s loop regulates the specificity and cross-reactivity of ligands for their cognate death receptors (55). It is tempting to speculate that α2-6 and α2-3 sialylation on these sites may have divergent effects on TNFR1/Fas structure, given the small size of the 90s loop and crucial positioning of the loop within the ligand-binding pocket. The bonds linking α2-6 and α2-3 sialic acids to galactose are spatially distinct, therefore these two linkages would be expected to place the sialic acid in different positions within the vicinity of the 90s loop. *In silico* studies have suggested that the Fas *N*-glycans are in key positions to modulate both Fas trimerization and higher-order oligomerization (48). In particular, N136 is predicted to lie within the interface between the three Fas protomers comprising the trimer (56), where it likely forms hydrogen bonds with the ligand for Fas, FasL (48). Given that sialic acid is a bulky, negatively-charged sugar, its spatial position relative to the polypeptide backbone could have a significant influence on various aspects of protein structure including conformation and oligomerization.

Independent of the effects of sialylation on TNFR1 and Fas structure, the two sialic acid linkages may differentially regulate apoptosis by modulating receptor binding to the extracellular galectin lattice. Galectins are galactose-binding lectins that can induce the clustering and activation of various receptors including Fas. Many groups have shown that the addition of α2-6, but not α2-3, sialylation to galactose on *N*-glycans inhibits the binding of most galectins (57). Hirabayashi *et al.* reported that galectin recognition of galactose required a free hydroxyl on the sixth carbon of galactose, which is the site for α2-6 sialylation (58). The third carbon of galactose, which is the site for α2-3 sialylation, was not involved in galectin binding (58). These data offer a mechanistic explanation for why α2-6, but not α2-3, sialylation generally inhibits galectin binding and function. Galectin-1 is known to bind directly to Fas to induce apoptosis (59). It was proposed that the binding of galectin-1 was sufficient to induce ligand-independent clustering of Fas, leading to the recruitment and activation of caspase 8 (60). The binding of galectin-3 to Fas has likewise been shown to promote caspase activation (61). In contrast to Fas, little is known regarding galectin-dependent regulation of apoptotic signaling by TNFR1. However, the studies of galectin/Fas interactions highlight another plausible mechanism by which the distinct sialic acid linkages might have variable effects on death receptor activity.

A role for sialylation in death receptor signaling was first identified nearly 3 decades ago. In 1995, Peter *et al.* reported that high levels of sialylation on Fas were correlated with reduced Fas-dependent caspase activation, and the enzymatic removal of sialic acids restored caspase activity (62). Subsequent studies have confirmed that treating cells with a broad spectrum sialidase leads to more robust Fas and TNFR1-mediated cell death (29, 63). Congruently, deletion of the CMAS gene, which synthesizes the CMP-sialic acid substrate, activates Fas-mediated apoptosis in B cells (64). While these findings affirm the importance of sialylation in death receptor signaling, a caveat is that the methods utilized in these prior studies eliminated all surface sialic acids. Complete loss of surface sialylation can occur biologically, for instance, through sialidases produced by pathogens or upregulation of certain members of the Neu family of mammalian neuraminidases (65, 66, 67). However, in cancer cells, changes in the composition of sialoglycans, including alterations in the sialic acid linkage, are more common than a discrete switch between an unsialylated and sialylated state. Cancer cells are generally hypersialylated, and increases in α2-3, α2-6, and α2,8 sialylation have all been documented (68, 69). Nonetheless, there is evidence that α2-6 sialylated *N*-glycans may be selectively enriched in some types of malignancies (70, 71, 72, 73, 74, 75). Deciphering the disparate biological effects of the various sialic acid linkages is essential for understanding the contribution of specific sialyltransferases, including ST6GAL1, to pathogenesis.

The present investigation provides a conceptual advance by demonstrating that ST6GAL1-mediated α2-6 sialylation plays a selective role in regulating the clustering and internalization of TNFR1, thereby controlling the balance between survival and apoptotic signaling. We postulate that increased α2-6 sialylation of TNFR1 and Fas protects cancer cells from apoptosis induced by TNF and FasL, both of which are highly enriched within an inflammatory milieu (76, 77, 78, 79). Finally, this study paves the way for the development of therapeutic approaches aimed at targeting the α2-6 sialylation of TNFR1/Fas, which may hold potential for limiting the off-target effects associated with more generic strategies that modulate the expression or overall signaling activity of TNFR1 and Fas.

## Experimental procedures

### Cell culture

Engineered HEK293 cell lines (ΔST3, ΔST6) were generated by CRISPR/Cas-9 mediated KO of select sialyltransferase genes in wild-type HEK293 (WT) cells as described (40). Lentivirus encoding human *ST6GAL1* (GeneCopoeia, LPP-M0351-Lv197) was used to transduce ΔST6 cells to generate the ΔST6-R cell line, which has stable re-expression of ST6GAL1. Lentiviral transduction was performed using an MOI of 5, and stable polyclonal populations were selected using 10 μg/ml Blasticidin S HCl (Gibco, A1113903). For routine culture of the four cell lines, cells were grown in DMEM/High Glucose (Gibco, 11965092) with 10% fetal bovine serum (FBS) (Atlanta Biologicals), 1% GlutaMAX Supplement (Gibco, 35050079) and 1% Antibiotic-Antimycotic (Gibco, 15240062). Deletion and restoration of ST6GAL1 expression and activity were confirmed by immunoblotting and SNA staining. To induce TNFR1 activation, cells were pre-incubated for 2 h in serum-free media and then treated for varying time points with 100 ng/ml of TNF (R&D, 10291-TA) in serum-free media. TNFR1 function-blocking antibody H398 (Invitrogen, BMS106, 5 μg/ml) and Dyngo-4a (Selleckchem, S7163, 5 μM) were administered to cells 1 h prior to TNF treatment. For Fas activation, cells were pre-incubated for 1 h with 5 μg/ml cycloheximide (CHX) (Sigma-Aldrich, C4859) in serum-free media, followed by treatment for 5 h with 500 ng/ml of the CH11 Fas activating antibody (Sigma-Aldrich, 05–201).

### Immunoblotting

Cells were lysed using radio-immunoprecipitation assay (RIPA) buffer (Thermo Scientific, 89,900) supplemented with protease and phosphatase inhibitors (Thermo Scientific, 78,440). Total protein concentration was assessed by BCA (Thermo Scientific, 23,227). Proteins were suspended in lithium dodecyl sulfate (LDS) sample buffer (Invitrogen, NP0007) containing β-mercaptoethanol (BME) (Gibco, 21985023). Samples were resolved by SDS-PAGE, followed by transfer to polyvinylidene difluoride (PVDF) membranes (Millipore, IPVH00010). Membranes were blocked with 5% nonfat dry milk in TBS containing 0.1% Tween-20 (TBST) for 1 h. The membranes were then probed with antibodies against ST6GAL1 (1:300, R&D, AF5924), TNFR1 (1:500, Cell Signaling Technology, 3736), Fas (1:500, Cell Signaling Technology, 8023), cleaved caspase 8 (1:500, Cell Signaling Technology, 9496), cleaved caspase 3 (1:500, Cell Signaling Technology, 9664), phosphorylated NFκB p65 (pSer536, 1:1000, Cell Signaling Technology, 3033), total NFκB p65 (1:1000, Cell Signaling Technology, 8242), and A20/TNFAIP3 (1:1000, Cell Signaling Technology, 5630). Membranes were washed in TBST and then incubated with the appropriate secondary antibodies coupled to horseradish-peroxidase (HRP) (Cell Signaling Technologies). Equal protein loading was confirmed by blotting with an anti-β-actin antibody (1:1000, Invitrogen, MA1-140-A488). Membranes were developed with Clarity Western ECL Substrate (Bio-Rad, 1705061) or SuperSignal West Femto (Pierce, 34,096). Densitometric analyses were performed using Image J on at least 3 independent blots (*i.e.*, from three independently-generated cell lysates). Densitometric units (DUs) were normalized to β-actin. For immunoblots depicting NFκB activation, p-NFκB was first normalized to t-NFκB, and then the ratio was normalized to β-actin.

### Lectin precipitation (SNA, MAA, ECL)

500 μg of cell lysate was incubated with 50 μl of agarose-conjugated SNA (Vector Laboratories, AL-1303–2), agarose-conjugated MAA, or agarose-conjugated ECL (Vector Laboratories, AL-1143–2) on a rotator at 4 °C overnight. Agarose beads bound with α2-6 sialylated (SNA), α2-3 sialylated (MAA), or unsialylated (ECL) protein were precipitated by centrifugation and washed three times with PBS. Precipitates were then immunoblotted for TNFR1 or Fas as described above.

### Flow cytometry

Cells were detached by Accutase (Corning, 25–058-CI), washed in PBS, and blocked for 30 min on ice in PBS containing 1% BSA. Cells were then incubated for 30 min on ice with the following antibodies: anti-TNFR1-Alexa 488 (1:100, Santa Cruz, sc-8436 AF488), or anti-Fas-APC (1:20, Invitrogen, 17–0959–42). Cells were also stained for 30 min on ice with lectins including SNA-Cy5 (1:200, Vector Laboratories, CL-1305–1), MAA-FITC (1:200, EY Laboratories, F-7801–2), SiaFind α2-3-Specific Lectenz SureLight 488 (1:400, Lectenz Bio, SK2301F), and ECL-fluorescein (1:250, Vector Laboratories, FL-1141–5). After staining, cells were washed three times with PBS. Cells were evaluated using the LSR Fortessa flow cytometer (BD Bioscience). Histograms were plotted using FlowJo version 10 software.

### TNFR1 internalization assay

Cells were treated for 60 min on ice with TNF containing a Flag tag at the N-terminus (Enzo Life Sciences, ALX-522–008). The incubation was performed on ice to allow Flag-TNF binding to surface TNFR1, while preventing the internalization of TNF-TNFR1 complexes. Additionally, we used a higher concentration of Flag-TNF, 500 ng/ml, as compared with the 100 ng/ml TNF concentration used for cell signaling assays because our goal was to maximize the binding of TNF to TNFR1. After the 60-min treatment of cells with Flag-TNF on ice, cells were washed and incubated at 37 °C for 30 min to induce the internalization of TNF-TNFR1 complexes. The internalization step was then terminated by placing cells back on ice. To measure the levels of surface TNF-TNFR1 complexes, cells were stained with anti-Flag-Alexa 647 (1:200, Invitrogen, 701629RP647) for 20 min on ice. Cells were stained with anti-Flag before internalization (*i.e.*, at the end of the 60-min incubation with Flag-TNF on ice) and then after the 30-min internalization step at 37 °C. Flow cytometry was performed, and mean fluorescence intensity (MFI) values were measured by FlowJo. The relative amount of internalization was calculated by subtracting MFI values obtained after the 37 °C internalization step from MFI values obtained at 4 °C prior to the internalization step. Results were normalized to the WT samples.

### Caspase 3/7 luminescence assay

Cells were seeded in equal numbers into 96-well tissue culture plates and allowed to adhere overnight. Cells were serum-deprived for 2 h and then treated with 100 ng/ml TNF for 5 h. For Fas-driven apoptosis, cells were pre-incubated for 1 hour with 5 μg/ml CHX in serum-free media, followed by a 5 h treatment with 500 ng/ml of the CH11 Fas-activating antibody (Sigma-Aldrich, 05–201). The Caspase-Glo 3/7 luminescence reagent (Promega, G8092) was added to each well, and cells were incubated for 45 min at room temperature with agitation on an orbital shaker. After this incubation, luminescence signals were measured by a BioTek Synergy H1 plate reader (Agilent). One another 96-well plate with the same condition as the plate for caspase 3/7 assay was utilized for CellTiter-Glo (ATP quantification) assay (Promega, G7572) as a representation of the overall cell number. The results are presented as a ratio of Caspase-Glo to CellTiter-Glo, and values were normalized for the untreated WT cell line.

### TIRF microscopy with fixed cells

The HEK293 cell lines were seeded overnight on #1.5 glass coverslips (Thorlabs, CG15XH) coated with poly-D-lysine (Gibco, A3890401). Cells were serum-starved for 2 h and then treated with or without 100 ng/ml TNF for 15 min at 37 °C. Cells were washed with ice-cold PBS to stop further TNF signaling and fixed with 4% paraformaldehyde (Electron Microscopy Services, 15,710). Fixed cells were washed thrice with PBS and blocked for 30 min at 37 °C with PBS containing 0.25% Triton X-100, 1% BSA, 5% horse serum (Gibco, 16050130), and 5% goat serum (Gibco, 16210064). Subsequently, cells were stained for 2 h at 37 °C with a primary antibody against TNFR1 (1:50, Santa Cruz, sc-8436). After staining, cells were washed three times with rinse buffer (0.5% horse serum, 0.5% goat serum, and 0.05% Triton X-100 in PBS) and then incubated for 1 hour at 37 °C with a secondary anti-mouse Alexa Fluor 488 antibody (1:500, Invitrogen, A32766). Cells were washed thrice with rinse buffer before imaging in FluoroBrite DMEM (Gibco, A1896701). To evaluate the distribution and clustering of TNFR1 on the cell membrane, cells were imaged using TIRF and RICM microscopy as previously described (80). Cells were imaged on a Nikon Eclipse Ti2 microscope using Nikon Elements software under TIRF and RICM modalities with an oil-immersion Apo TIRF 60 × NA 1.49 objective and an ORCA-Flash 4.0 V3 Digital CMOS camera (Hamamatsu). For TIRF imaging, a Nikon TIRF arm with a 488 nm laser (Coherent, OBIS) was used for illumination and emission passed through a 525/50 bandpass filter. For RICM imaging, the sample was illuminated with a Sola epifluorescence light source (Lumencor).

### BS^3^ cross-linking experiments

The cross-linking protocol was adapted from protocols developed by other groups (44, 45). Cells were incubated for 30 min on ice with 1 mM of BS^3^ (bis(sulfosuccinimidyl)suberate) (Thermo Scientific, 21,580). The reaction was quenched by adding Tris-HCl to a final concentration of 20 mM for 15 min. After washing with PBS, cells were lysed and immunoblotted for TNFR1, as described earlier. Quantification of the immunoblots was performed using densitometry by ImageJ. Results are represented as the ratio of TNFR1 oligomers (>250 kD) to monomers from three independent experiments.

### Time-lapse TIRF microscopy with live cells

The HEK293 cell lines were seeded overnight on poly-D-lysine-coated coverslips as above. Cells were transiently transfected for 24 h at 37 °C with the GFP-tagged TNFR1 plasmid (GeneCopoeia, EX-Z2785-M98, Accession: NM_001065.4) using Lipofectamine 3000 (Invitrogen, L3000001). Following transfection, HEK293 cells were serum-starved for 2 h before imaging. For time-lapse live cell TIRF microscopy, cells were in FluoroBrite DMEM (Gibco, A1896701), and image acquisition was performed using the same microscope and software as in the fixed-cell TIRF experiments with the addition of a stage-top incubator to maintain cells at 37 °C and 5% CO_2_ (Tokai Hit, INUBG2SF-TIZB). Imaging was conducted immediately before TNF treatment (0 min) and every 2 min following treatment with 100ng/ml of TNF for 30 min.

### Image processing and analysis

For the fixed cell TIRF experiments, custom-written ImageJ macros were used to conduct background subtraction. To define the cell boundary, we manually outlined the cell using the RICM image. We applied the cell boundary to the TIRF image to obtain the average intensity. The Analyze Particle function was employed after default auto-thresholding of the background-subtracted image to generate a mask for analyzing the characteristics of TNFR1 clusters. TNFR1 cluster diameter was determined using default parameters in the Analyze Particles function. The diameter of the TNFR1 cluster (μm) was calculated using the formula: $iameter\left(\mu m\right)=2\times \sqrt{\frac{cluster\;area\;\left({\mu m}^{2}\right)\;}{\pi }}$. To calculate cluster number, and the percentage of membrane occupancy, we used 9-pixel^2^ (∼0.1 μm^2^) to infinity for the “size” input.

For time-lapse live cell TIRF experiments, average intensity and proportion of membrane occupancy were calculated as described above. For comparative analysis, we normalized the values for the average intensity and proportion of membrane occupancy induced by TNF to the values for untreated cells (0 min).

### Protein structure illustration

TNF/TNFR1 complex structure (PDB: 7KP7) was illustrated in UCSF ChimeraX (81). FasL/Fas complex structure was generated by homology modeling in UCSF ChimeraX using FasL (PDB: 5L19), Fas (PDB: 3TJE), and TNF/TNFR1 (PDB: 7KP7) as reference structures.

### Statistical analyses

As indicated in the figure legends, statistical significance was evaluated by one-way or two-way ANOVA using GraphPad Prism (v. 10.0.2). At least 3 biological replicates were examined for all experiments, and data are presented as mean ± SD

## Data availability

All data described in this study are contained within the manuscript or supplementary figures and tables.

## Supporting information

This article contains supporting information.

## Conflict of interest

The authors declare that they have no conflicts of interest with the contents of this article.
