# Supplementary material for: Apoptotic signaling by TNFR1 is inhibited by the α2-6 sialylation, but not α2-3 sialylation, of the TNFR1 N-glycans
Source: J Biol Chem. 2024 Nov 29;301(1):108043. doi: 10.1016/j.jbc.2024.108043 (PMC11732462; doi:10.1016/j.jbc.2024.108043)
Supplement: Supplementary_Figures [file mmc1.pdf]

Apoptotic signaling by TNFR1 is inhibited by the α2-6 sialylation, but not α2-3 sialylation, of the TNFR1 *N*-glycans

Jihye Hwang[1], Tejeshwar C. Rao[1], Jiahui Tao[1], Bingdong Sha[1], Yoshiki Narimatsu[2], Henrik Clausen[2], Alexa L. Mattheyses[1]*, Susan L. Bellis[1]*

[1]Department of Cell, Developmental and Integrative Biology, University of Alabama at Birmingham, Birmingham, AL

[2]Copenhagen Center for Glycomics, Department of Cellular and Molecular Medicine, University of Copenhagen, Copenhagen, DK-2200 N, Denmark

***To whom correspondence should be addressed:**
Susan L. Bellis, Ph.D.
Department of Cell, Developmental and Integrative Biology
University of Alabama at Birmingham
Birmingham, AL 35294
(205) 934-3441
bellis@uab.edu

Alexa L. Mattheyses, Ph.D.
Department of Cell, Developmental and Integrative Biology
University of Alabama at Birmingham
Birmingham, AL 35294
(205) 975-0680
mattheyses@uab.edu

Running title: α2-6, but not α2-3, sialylation of TNFR1 inhibits apoptosis

Material Included:

Figure S1. Effects of TNF on surface TNFR1 clustering as measured by TIRF microscopy.
Figure S2. Live cell TIRF images every two minutes following TNF stimulation.
Figure S3. Statistical analysis of live cell TIRF for intensity in TNF-treated vs. untreated cells.
Figure S4. Statistical analysis of live cell TIRF for membrane occupancy in TNF-treated vs. untreated cells.
Table S1. Statistical analysis of live cell TIRF for intensity comparing WT, ΔST3, ΔST6 and ΔST6-R cells.
Table S2. Statistical analysis of live cell TIRF for membrane occupancy comparing WT, ΔST3, ΔST6 and ΔST6-R cells.

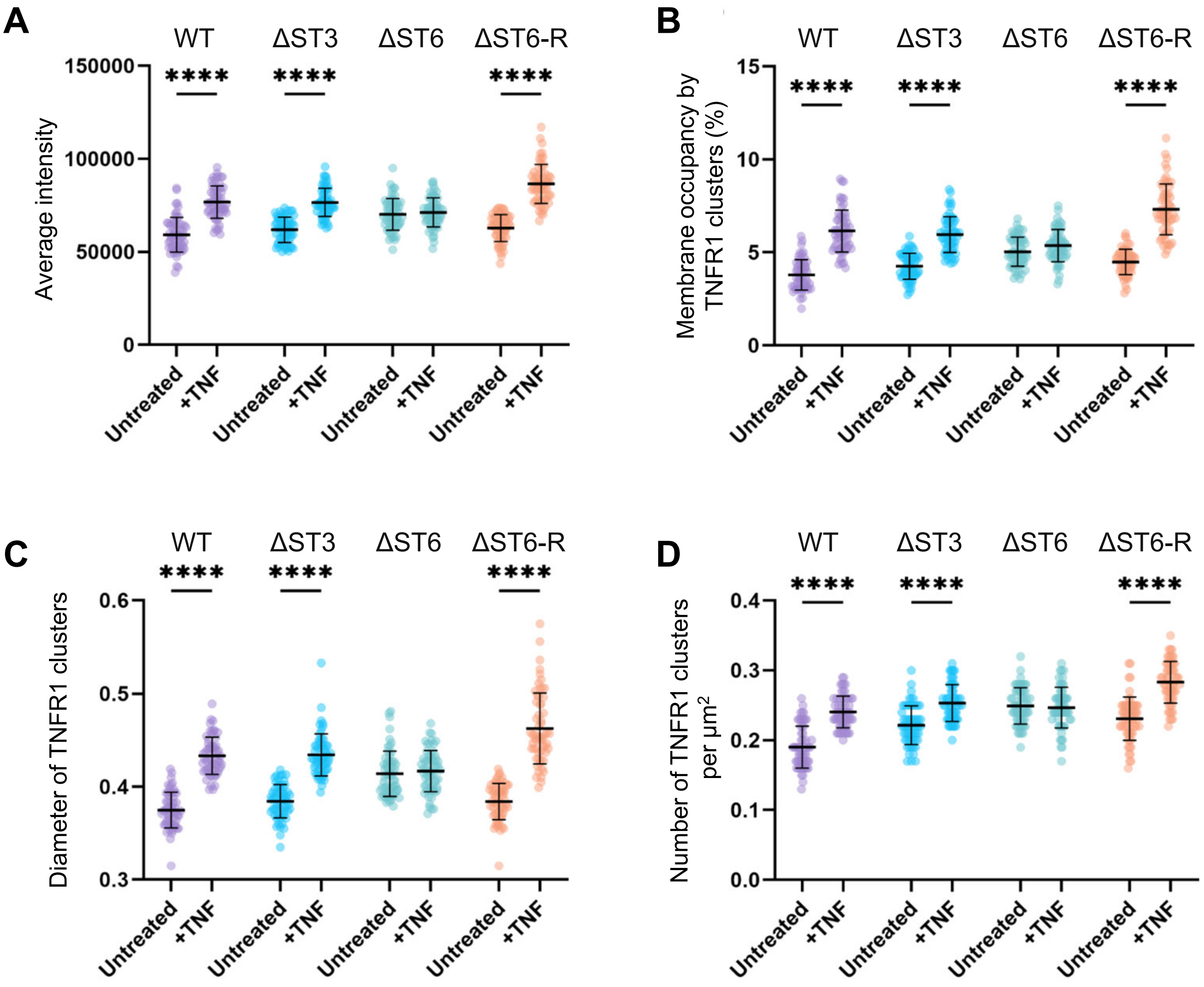

Figure S1. Effects of TNF on surface TNFR1 clustering as measured by TIRF microscopy. HEK293 cells were treated with or without 100 ng/mL TNF for 15 minutes. Cells were then fixed and stained for TNFR1. TIRF microscopy was used to evaluate TNFR1 clusters on the cell surface. A) Average intensity per image (measured as arbitrary fluorescence units per μm^2). B) Percentage of the cell membrane occupied by TNFR1 clusters. C) Diameter of TNFR1 clusters per image (μm). D) Number of TNFR1 clusters per μm^2. Panels A-D show that TNF treatment induced changes in TNFR1 clustering parameters for all cell lines except ΔST6 cells. Graphs represent mean ± S.D. from two independent experiments, with 60 cells analyzed per group. Statistical analysis was performed using two-way ANOVA with Tukey's multiple comparisons test (****: $p < 0.0001$).

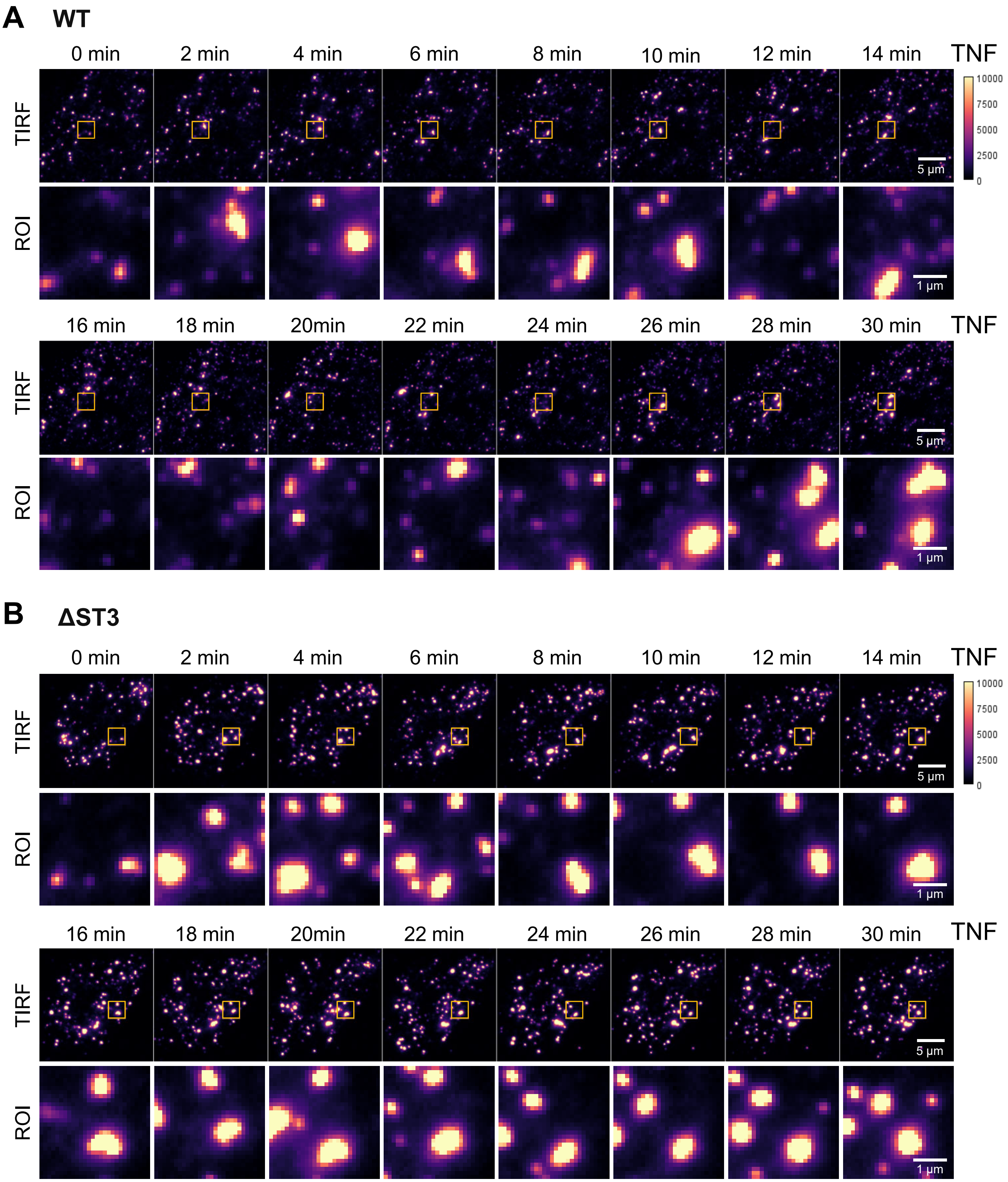

Figure S2. Live cell TIRF images every two minutes following TNF stimulation.
Cells were transfected with TNFR1-GFP to enable live cell TIRF microscopy. TIRF images were taken immediately before treatment (0 minutes) and then every 2 minutes after treatment with 100 ng/mL TNF. Representative images of WT HEK293 cells (A) and ΔST3 cells (B).

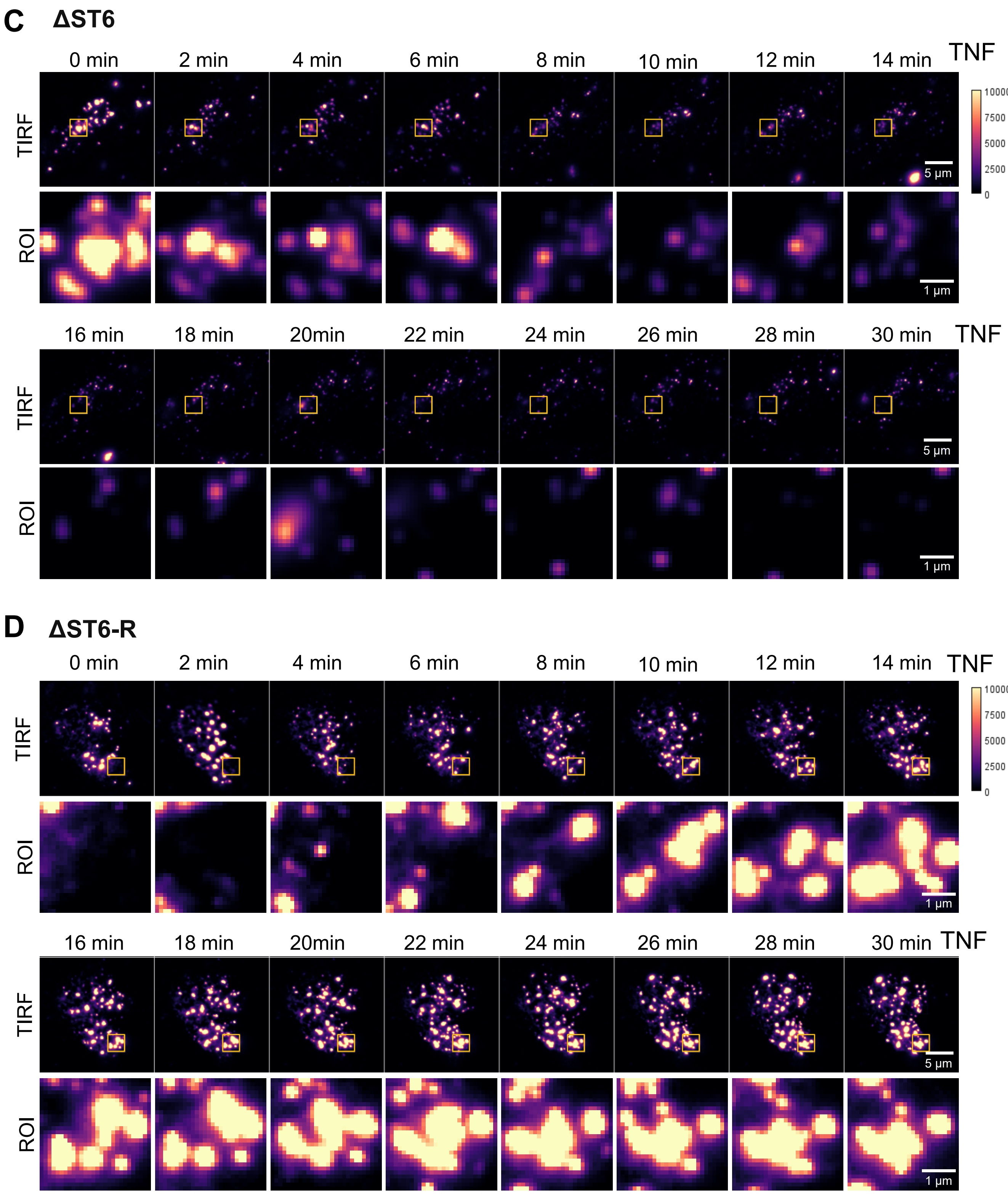

Figure S2. Live cell TIRF images every two minutes following TNF stimulation (continued) Representative images of ΔST6 cells (C) and ΔST6-R cells (D). No discernable differences were observed in WT, ΔST3, and ΔST6-R cells, whereas a marked loss in surface TNFR1 clusters was noted in ΔST6 cells. Scale bars represent 5 μm for TIRF and 1 μm for the region of interest (ROI).

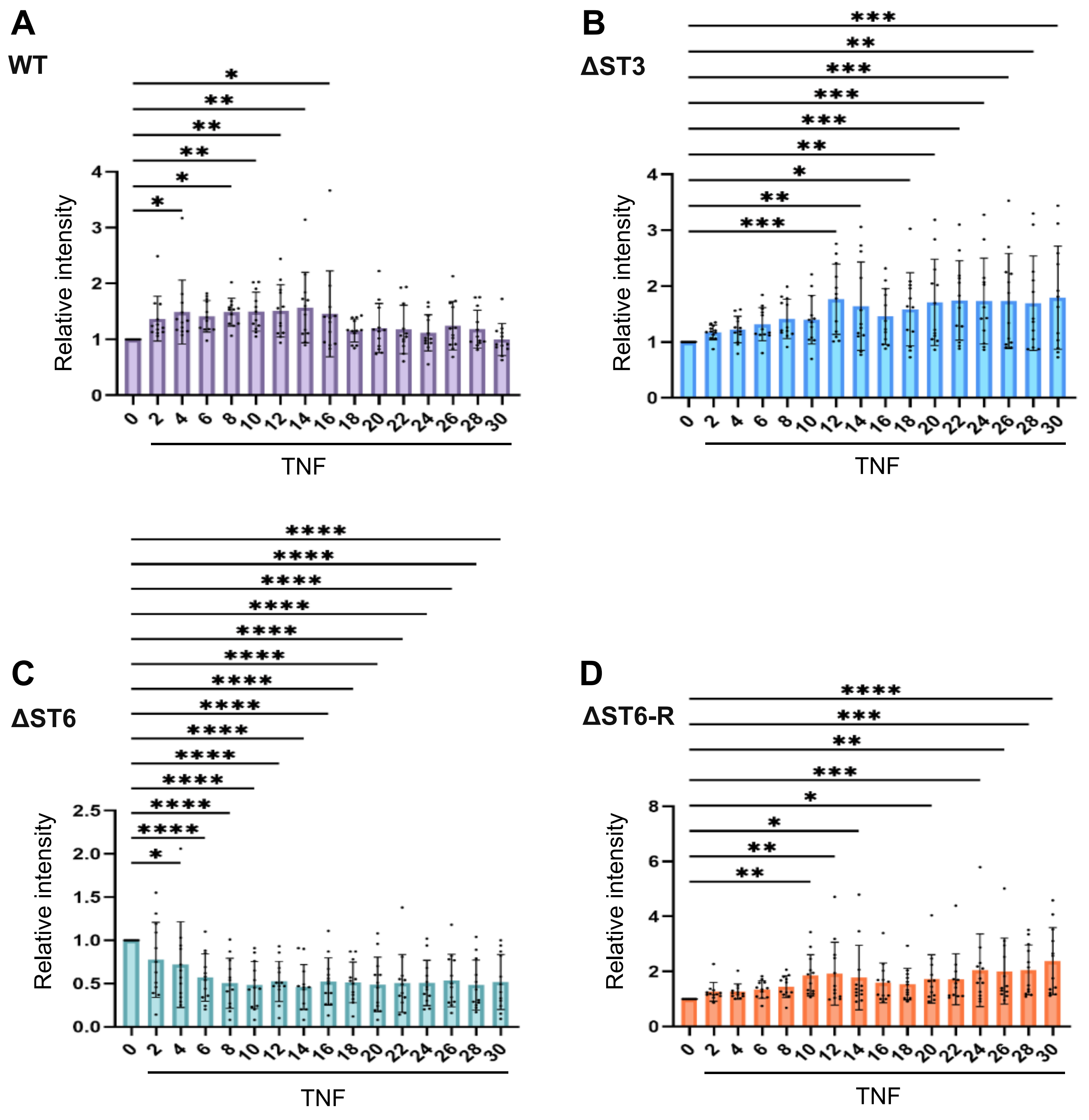

Figure S3. Statistical analysis of live cell TIRF for intensity in TNF-treated vs. untreated cells. Live cell TIRF imaging was employed to analyze the relative intensity of cells transfected with TNFR1-GFP. Cells were imaged immediately before TNF treatment (0 minutes) and every 2 minutes after treatment with 100 ng/mL TNF. Values for TNF-treated cells were normalized to the untreated values. A) WT cells. B) ΔST3 cells. C) ΔST6 cells. D) ΔST6-R cells. Panels A-D show that TNF treatment increased the relative intensity for WT, ΔST3, and ΔST6-R cells, whereas a reduction in relative intensity was noted for ΔST6 cells. Graphs depict mean ± S.D. Data were analyzed by one-way ANOVA followed by Dunnett's multiple comparisons test (*: $p < 0.05$, **: $p < 0.01$, ***: $p < 0.001$, ****: $p < 0.0001$).

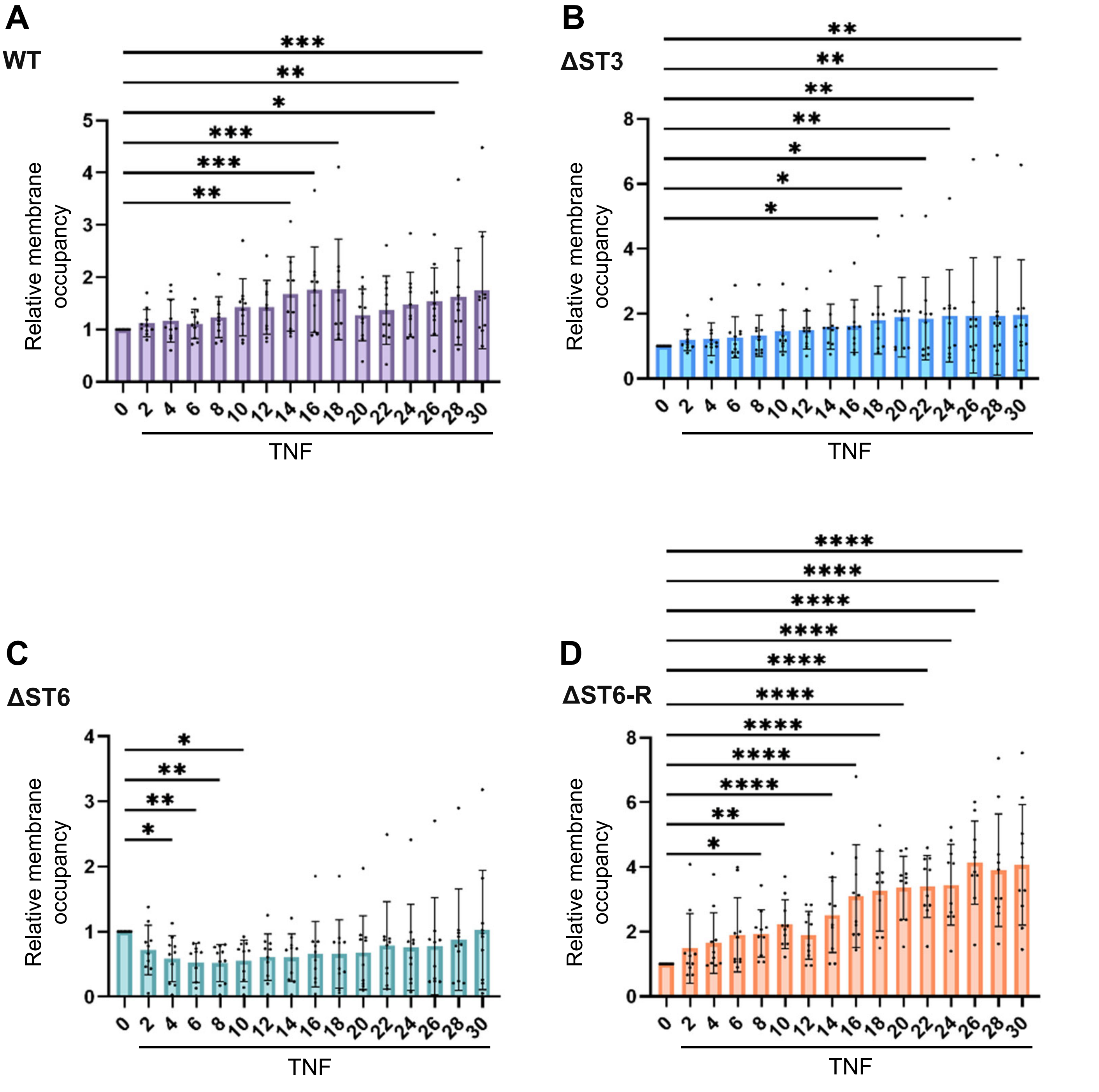

Figure S4. Statistical analysis of live cell TIRF for membrane occupancy in TNF-treated vs. untreated cells.
Live cell TIRF imaging was employed to analyze the relative proportion of the plasma membrane occupied by TNFR1-GFP clusters. Cells were imaged immediately before TNF treatment (0 minutes) and every 2 minutes after treatment with 100 ng/mL TNF. Values for TNF-treated cells were normalized to the untreated values. A) WT cells. B) ΔST3 cells. C) ΔST6 cells. D) ΔST6-R cells. Panels A-D show that TNF increased the membrane occupancy by TNFR1-GFP for WT, ΔST3, and ΔST6-R cells, whereas a reduction in occupancy was noted for ΔST6 cells. Graphs depict mean ± S.D. Data were analyzed by one-way ANOVA followed by Dunnett's multiple comparisons test (*: $p < 0.05$, **: $p < 0.01$, ***: $p < 0.001$, ****: $p < 0.0001$).

Table S1: Statistical analysis of live cell TIRF for intensity comparing WT, ΔST3, ΔST6 and ΔST6-R cells.

.

Group comparison	Below threshold?	Summary	Adjusted P Value
2 minutes TNF			
WT vs. ΔST3	No	ns	0.4002
WT vs. ΔST6	Yes	*	0.0103
WT vs. ΔST6-R	No	ns	0.8827
ΔST3 vs. ΔST6	Yes	*	0.0414
ΔST3 vs. ΔST6-R	No	ns	0.849
ΔST6 vs. ΔST6-R	Yes	*	0.0299
4 minutes TNF			
WT vs. ΔST3	No	ns	0.4715
WT vs. ΔST6	Yes	**	0.0098
WT vs. ΔST6-R	No	ns	0.6463
ΔST3 vs. ΔST6	Yes	*	0.0272
ΔST3 vs. ΔST6-R	No	ns	0.9674
ΔST6 vs. ΔST6-R	Yes	*	0.017
6 minutes TNF			
WT vs. ΔST3	No	ns	0.8592
WT vs. ΔST6	Yes	****	<0.0001
WT vs. ΔST6-R	No	ns	0.9427
ΔST3 vs. ΔST6	Yes	****	<0.0001
ΔST3 vs. ΔST6-R	No	ns	0.9974
ΔST6 vs. ΔST6-R	Yes	****	<0.0001
8 minutes TNF			
WT vs. ΔST3	No	ns	0.9325
WT vs. ΔST6	Yes	****	<0.0001
WT vs. ΔST6-R	No	ns	0.9935
ΔST3 vs. ΔST6	Yes	****	<0.0001
ΔST3 vs. ΔST6-R	No	ns	0.9943
ΔST6 vs. ΔST6-R	Yes	****	<0.0001
10 minutes TNF			
WT vs. ΔST3	No	ns	0.9297
WT vs. ΔST6	Yes	****	<0.0001
WT vs. ΔST6-R	No	ns	0.4463
ΔST3 vs. ΔST6	Yes	****	<0.0001
ΔST3 vs. ΔST6-R	No	ns	0.2865
ΔST6 vs. ΔST6-R	Yes	***	0.0002
12 minutes TNF			
WT vs. ΔST3	No	ns	0.6729
WT vs. ΔST6	Yes	****	<0.0001
WT vs. ΔST6-R	No	ns	0.6662
ΔST3 vs. ΔST6	Yes	****	<0.0001
ΔST3 vs. ΔST6-R	No	ns	0.9765
ΔST6 vs. ΔST6-R	Yes	**	0.0066
14 minutes TNF			
WT vs. ΔST3	No	ns	0.9951
WT vs. ΔST6	Yes	***	0.0003
WT vs. ΔST6-R	No	ns	0.9459
ΔST3 vs. ΔST6	Yes	**	0.0013
ΔST3 vs. ΔST6-R	No	ns	0.9854
ΔST6 vs. ΔST6-R	Yes	*	0.0112
16 minutes TNF			
WT vs. ΔST3	No	ns	>0.9999
WT vs. ΔST6	Yes	**	0.0071
WT vs. ΔST6-R	No	ns	0.9727
ΔST3 vs. ΔST6	Yes	***	0.0001
ΔST3 vs. ΔST6-R	No	ns	0.9549
ΔST6 vs. ΔST6-R	Yes	**	0.0014
18 minutes TNF			
WT vs. ΔST3	No	ns	0.1877
WT vs. ΔST6	Yes	****	<0.0001
WT vs. ΔST6-R	No	ns	0.177
ΔST3 vs. ΔST6	Yes	***	0.0006
ΔST3 vs. ΔST6-R	No	ns	0.998
ΔST6 vs. ΔST6-R	Yes	***	0.0002
20 minutes TNF			
WT vs. ΔST3	No	ns	0.2341
WT vs. ΔST6	Yes	***	0.001
WT vs. ΔST6-R	No	ns	0.2838
ΔST3 vs. ΔST6	Yes	***	0.0008
ΔST3 vs. ΔST6-R	No	ns	>0.9999
ΔST6 vs. ΔST6-R	Yes	**	0.0021
22 minutes TNF			
WT vs. ΔST3	No	ns	0.1189
WT vs. ΔST6	Yes	**	0.0019
WT vs. ΔST6-R	No	ns	0.3003
ΔST3 vs. ΔST6	Yes	***	0.0003
ΔST3 vs. ΔST6-R	No	ns	0.9998
ΔST6 vs. ΔST6-R	Yes	**	0.004
24 minutes TNF			
WT vs. ΔST3	No	ns	0.0924
WT vs. ΔST6	Yes	***	0.0003
WT vs. ΔST6-R	No	ns	0.1393
ΔST3 vs. ΔST6	Yes	***	0.0007
ΔST3 vs. ΔST6-R	No	ns	0.8959
ΔST6 vs. ΔST6-R	Yes	**	0.0092
26 minutes TNF			
WT vs. ΔST3	No	ns	0.3153
WT vs. ΔST6	Yes	***	0.0008
WT vs. ΔST6-R	No	ns	0.2038
ΔST3 vs. ΔST6	Yes	**	0.0022
ΔST3 vs. ΔST6-R	No	ns	0.9127
ΔST6 vs. ΔST6-R	Yes	**	0.0061
28 minutes TNF			
WT vs. ΔST3	No	ns	0.2534
WT vs. ΔST6	Yes	***	0.0001
WT vs. ΔST6-R	Yes	*	0.0351
ΔST3 vs. ΔST6	Yes	**	0.0019
ΔST3 vs. ΔST6-R	No	ns	0.7529
ΔST6 vs. ΔST6-R	Yes	***	0.0004
30 minutes TNF			
WT vs. ΔST3	No	ns	0.0578
WT vs. ΔST6	Yes	**	0.0042
WT vs. ΔST6-R	Yes	*	0.0113
ΔST3 vs. ΔST6	Yes	**	0.0025
ΔST3 vs. ΔST6-R	No	ns	0.5616
ΔST6 vs. ΔST6-R	Yes	**	0.0011

Statistical comparison of live cell TIRF results for TNFR1-GFP intensity comparing WT, ΔST3, ΔST6, and ΔST6-R cells at each time point following treatment with 100 ng/mL of TNF. Notably, values for the ΔST6 cells were statistically different from all other cell lines at every time point. Data were analyzed by two-way ANOVA followed by Tukey’s multiple comparisons test.

Table S2: Statistical analysis of live cell TIRF for membrane occupancy comparing WT, ΔST3, ΔST6 and ΔST6-R cells.

Group comparison	Below threshold?	Summary	Adjusted P Value
2 minutes TNF			
WT vs. ΔST3	No	ns	0.9472
WT vs. ΔST6	No	ns	0.0607
WT vs. ΔST6-R	No	ns	0.7272
ΔST3 vs. ΔST6	Yes	*	0.0368
ΔST3 vs. ΔST6-R	No	ns	0.8412
ΔST6 vs. ΔST6-R	No	ns	0.201
4 minutes TNF			
WT vs. ΔST3	No	ns	0.9958
WT vs. ΔST6	Yes	*	0.0148
WT vs. ΔST6-R	No	ns	0.4701
ΔST3 vs. ΔST6	Yes	*	0.0237
ΔST3 vs. ΔST6-R	No	ns	0.5817
ΔST6 vs. ΔST6-R	Yes	*	0.0251
6 minutes TNF			
WT vs. ΔST3	No	ns	0.8659
WT vs. ΔST6	Yes	**	0.0015
WT vs. ΔST6-R	No	ns	0.2068
ΔST3 vs. ΔST6	Yes	*	0.0219
ΔST3 vs. ΔST6-R	No	ns	0.4544
ΔST6 vs. ΔST6-R	Yes	*	0.0179
8 minutes TNF			
WT vs. ΔST3	No	ns	0.9816
WT vs. ΔST6	Yes	**	0.0011
WT vs. ΔST6-R	No	ns	0.0727
ΔST3 vs. ΔST6	Yes	*	0.0139
ΔST3 vs. ΔST6-R	No	ns	0.2142
ΔST6 vs. ΔST6-R	Yes	***	0.0005
10 minutes TNF			
WT vs. ΔST3	No	ns	0.9988
WT vs. ΔST6	Yes	**	0.0029
WT vs. ΔST6-R	No	ns	0.0631
ΔST3 vs. ΔST6	Yes	**	0.0062
ΔST3 vs. ΔST6-R	No	ns	0.103
ΔST6 vs. ΔST6-R	Yes	***	0.0001
12 minutes TNF			
WT vs. ΔST3	No	ns	0.9898
WT vs. ΔST6	Yes	**	0.0039
WT vs. ΔST6-R	No	ns	0.3925
ΔST3 vs. ΔST6	Yes	**	0.0048
ΔST3 vs. ΔST6-R	No	ns	0.5776
ΔST6 vs. ΔST6-R	Yes	**	0.0014
14 minutes TNF			
WT vs. ΔST3	No	ns	0.9933
WT vs. ΔST6	Yes	**	0.004
WT vs. ΔST6-R	No	ns	0.2549
ΔST3 vs. ΔST6	Yes	**	0.0064
ΔST3 vs. ΔST6-R	No	ns	0.1858
ΔST6 vs. ΔST6-R	Yes	**	0.0021
16 minutes TNF			
WT vs. ΔST3	No	ns	0.9802
WT vs. ΔST6	Yes	*	0.0119
WT vs. ΔST6-R	No	ns	0.1297
ΔST3 vs. ΔST6	Yes	*	0.0267
ΔST3 vs. ΔST6-R	No	ns	0.0839
ΔST6 vs. ΔST6-R	Yes	**	0.0035

Group comparison	Below threshold?	Summary	Adjusted P Value
18 minutes TNF			
WT vs. ΔST3	No	ns	0.9998
WT vs. ΔST6	Yes	*	0.0287
WT vs. ΔST6-R	Yes	*	0.0367
ΔST3 vs. ΔST6	Yes	*	0.0354
ΔST3 vs. ΔST6-R	Yes	*	0.0493
ΔST6 vs. ΔST6-R	Yes	***	0.0003
20 minutes TNF			
WT vs. ΔST3	No	ns	0.4883
WT vs. ΔST6	No	ns	0.0899
WT vs. ΔST6-R	Yes	***	0.0002
ΔST3 vs. ΔST6	No	ns	0.0601
ΔST3 vs. ΔST6-R	Yes	*	0.0391
ΔST6 vs. ΔST6-R	Yes	****	<0.0001
22 minutes TNF			
WT vs. ΔST3	No	ns	0.7183
WT vs. ΔST6	No	ns	0.2418
WT vs. ΔST6-R	Yes	***	0.0002
ΔST3 vs. ΔST6	No	ns	0.1402
ΔST3 vs. ΔST6-R	Yes	*	0.032
ΔST6 vs. ΔST6-R	Yes	****	<0.0001
24 minutes TNF			
WT vs. ΔST3	No	ns	0.7831
WT vs. ΔST6	No	ns	0.0912
WT vs. ΔST6-R	Yes	**	0.0029
ΔST3 vs. ΔST6	No	ns	0.13
ΔST3 vs. ΔST6-R	No	ns	0.0884
ΔST6 vs. ΔST6-R	Yes	***	0.0002
26 minutes TNF			
WT vs. ΔST3	No	ns	0.8997
WT vs. ΔST6	No	ns	0.1075
WT vs. ΔST6-R	Yes	***	0.0003
ΔST3 vs. ΔST6	No	ns	0.2724
ΔST3 vs. ΔST6-R	Yes	*	0.0278
ΔST6 vs. ΔST6-R	Yes	****	<0.0001
28 minutes TNF			
WT vs. ΔST3	No	ns	0.9673
WT vs. ΔST6	No	ns	0.2341
WT vs. ΔST6-R	Yes	*	0.0129
ΔST3 vs. ΔST6	No	ns	0.3751
ΔST3 vs. ΔST6-R	No	ns	0.0968
ΔST6 vs. ΔST6-R	Yes	**	0.0013
30 minutes TNF			
WT vs. ΔST3	No	ns	0.9878
WT vs. ΔST6	No	ns	0.4168
WT vs. ΔST6-R	Yes	*	0.02
ΔST3 vs. ΔST6	No	ns	0.4522
ΔST3 vs. ΔST6-R	No	ns	0.0722
ΔST6 vs. ΔST6-R	Yes	**	0.0023

Statistical comparison of live cell TIRF results for TNFR1-GFP membrane occupancy comparing WT, ΔST3, ΔST6, and ΔST6-R cells at each time point following treatment with 100 ng/mL of TNF. Values for the ΔST6 cells were statistically different from the other cell lines at most of the time points. Data were analyzed by two-way ANOVA followed by Tukey’s multiple comparisons test.
